# Treatment of radius or ulna fractures in the elderly: A systematic review covering effectiveness, safety, economic aspects and current practice

**DOI:** 10.1371/journal.pone.0214362

**Published:** 2019-03-28

**Authors:** Cecilia Mellstrand Navarro, Agneta Brolund, Carl Ekholm, Emelie Heintz, Emin Hoxha Ekström, Per Olof Josefsson, Lina Leander, Peter Nordström, Lena Zidén, Karin Stenström

**Affiliations:** 1 Department of Hand Surgery, Karolinska Institute, Institution for Clinical Research and Education, Södersjukhuset Hospital, Stockholm, Sweden; 2 Swedish Agency for Health Technology Assessment and Assessment of Social Services, Stockholm, Sweden; 3 Department of Orthopaedics, Sahlgrenska University Hospital, Gothenburg, Mölndal, Sweden; 4 Department of Learning, Informatics, Management and Ethics (LIME), Karolinska Institutet, Stockholm, Sweden; 5 Department of Orthopaedics, Skane University Hospital, Malmö, Sweden; 6 Department of Community Medicine and Rehabilitation, Geriatrics, Umeå, Sweden; 7 Department of Health and Rehabilitation, The Sahlgrenska Academy at the University of Gothenburg, Institute of Neuroscience and Physiology, Gothenburg, Sweden; University of Milano-Bicocca, ITALY

## Abstract

**Background:**

The objective of the present study was to evaluate effectiveness, complications and cost-effectiveness of any surgical or non-surgical treatment for radius or ulna fractures in elderly patients. Secondary objectives were to analyze present treatment traditions of distal radius fractures (DRF) in Sweden and to calculate resource usage for its treatment.

**Methods and findings:**

The assessment contains a systematic review of clinical and health economic studies comparing treatment options for radius or ulna fractures. The results regarding the effectiveness of the treatments are summarized in meta-analyses. In addition, the assessment contains a cost analysis for different treatment options commonly used for DRF care, and an analysis of registry data on the incidence and treatment of DRF. In total 31 randomized controlled trials were included in meta-analyses. When comparing functional outcome for plate fixation versus non-surgical treatment for DRF, there were no clinically important differences at one-year follow-up (mean difference [MD], -3.29, 95% CI, -7.03; 0.44). Similar results were found when comparing plating and percutaneous methods with respect to functional outcome (standardized mean difference [SMD], -0.07, 95% CI, -0.21; 0.07) and grip strength (MD, -3.47, 95% CI, -11.21; 4.28). There were no differences for minor complications, (risk difference [RD], -0.01, 95% CI, -0.07; 0.05) whereas major complications were less common for the percutaneous group, (RD, 0.02, 95% CI, 0.02; 0.03). Given the low number of studies, the evidence above was rated as moderate certainty. The cost for plate fixation versus plaster cast was estimated to 1698 compared to 137 US dollars. For DRF, plate fixation increased in Sweden between 2005 and 2013, and was the most common surgical method in 2013.

**Conclusions:**

Surgical treatment of moderately displaced distal radius fractures in elderly patients offers no clear benefit compared to non-surgical treatment. Plating procedures have become more common during the second millennium and involve higher costs and higher risk of major complications than percutaneous options.

## Introduction

Fractures of the distal radius (DRF) are among the most common fractures in elderly people, and the ulna is often also affected. Many studies have been performed comparing clinical outcome after treatment. Although these reports have added to our knowledge, optimal treatment for distal radius and ulna fractures is still unknown or debated. The evidence supporting the use of surgical treatment for DRF is limited [1, 2]. Nevertheless, many authors report rising incidences of surgical treatment of distal radius fractures, more specifically surgical treatment with plate fixation [3–6]. The lack of unequivocal results from previous studies could partly be explained by studies being under-powered and study population heterogeneous with regards to fracture patterns and patient age [7]. Numerous literature reviews have been performed for comparison of treatment methods for DRFs [8] but only few regard populations of higher ages [1, 2].

In this context, the primary aim of this study was to assess the effectiveness and cost-effectiveness of treatment options for fractures of the radius or ulna in a population with a mean age above 60 years by means of a literature review and consequent meta-analysis. Secondary aims were to perform an analysis of resource usage for treatment of radius fractures, and to investigate the Swedish national incidence rates of distal radius fractures and their treatments.

## Materials and methods

The assessment contains a systematic review of clinical and health economic studies comparing treatment options for fractures of the radius or ulna. The results regarding the effectiveness of the treatments are summarized in meta-analyses. In addition, the assessment contains a cost analysis for different treatment options commonly used for DRF care in a Swedish setting, and an analysis of registry data on the incidence and treatment of distal radius fractures in Sweden. All costs were converted to US dollars in 2016 using the method recommended by The Cochrane and Campbell Economic Methods Group, i.e. with PPPs (purchasing power parity) via The CCEMG–EPPI-Centre Cost Converter (v.1.5 last update: 29 April 2016) http://eppi.ioe.ac.uk/costconversion/ (IMF-PPP). This study was approved by the regional ethics board in Umeå and by the National Board of Health and Welfare in Sweden. We have only used data from published articles and anonymous registry data presented on a group level.

### Systematic review and meta-analysis

#### Protocol and registration

The present systematic review and meta-analysis was based upon studies published in peer-reviewed journals investigating benefits and possible risks of different methods for treating radius or ulna fractures in the elderly (mean age of at least 60 years). The age limit was selected with the purpose to include studies regarding an osteoporotic population. This review was conducted and funded within the framework for the Swedish Agency for Health Technology Assessment and Assessment of Social Services, SBU (www.sbu.se/en), a public agency which conducts health technology assessments. Methods of analysis and inclusion criteria for the project were specified in advance, as a part of the internal process at SBU. No protocol has been published.

#### Eligibility criteria

The criteria for eligibility included the following characteristics, PICOS; Population, Intervention, Comparator, Outcomes and Study design.

**Population**. Mean age of the study population 60 years or above. All study participants were treated for radius and/or ulna fracture. Studies on cadavers were excluded.

**Interventions.** Any operative or non-operative fracture treatment.

**Comparator**. Any comparator (e.g., any alternative treatment, operative or non-operative).

**Outcome and measures**. Functional outcomes, grip strength, adverse effects/complications, quality of life (QoL), costs and cost-effectiveness. Any validated measure was acceptable.

**Study design**. Randomized controlled trial (RCT), non-randomized controlled studies (Non-R) and comparative registry studies.

**Setting.** Any setting.

**Language.** Studies published in English and/or in Scandinavian languages.

**Publication type.** Studies published in peer-reviewed journals.

#### Information sources

Studies were identified by searching electronic databases, and by scanning the reference lists of either studies meeting the eligibility criteria or relevant systematic reviews. The electronic databases PubMed, Embase, Cochrane Library, and Scopus were searched from 1990 up to December 2016.

#### Search strategy

Electronic searches were conducted using a combination of medical subject headings (MeSH) and relevant text word terms related to old age, any fracture of the upper extremity and interventions and study type. A specific search filter for health economic studies was used. (For detailed information about the search strategies, see S1 Appendix.)

#### Study selection

Two reviewers (in total three pairs of expert senior scientists) independently screened the titles and abstracts for eligibility. All abstracts were screened using the online available scanning tool Rayyan [9]. All publications of potential relevance according to the inclusion criteria were retrieved in full text. Eligibility for inclusion was independently assessed by two reviewers. Disagreements were resolved by consensus. Reference lists of studies meeting the eligibility criteria and of relevant systematic reviews were screened for additional relevant studies. (For detailed information about the included studies, see S2 Appendix, excluded studies, see S3 Appendix.)

#### Risk of bias in individual studies

To determine the internal validity of the eligible trials, a pair of reviewers independently assessed the risk of bias according to the SBU checklists [10]. The checklists for assessing the risk of bias in the RCTs are based on the CONSORT statement and discloses risk of bias related to six main aspects: selection; treatment (including blinding); measurement; attrition; reporting; conflicts of interest [11]. The checklist was used to reveal shortcomings of the studies. The reviewers thereafter made an assessment of the extent to which the internal validity of the results could have been affected by these shortcomings.

A rating of low, moderate or high risk of bias was given to each category of items.

Based on the severity of the combined threats to internal validity, an overall rating of risk of bias was then given to each study. The principal sources of bias on which the overall ratings were based are presented in the results section. For the health economic studies, a specific check-list for within trial cost-effectiveness studies was used [12]. Only studies with a low or moderate overall risk of bias were included in the synthesis.

#### Data items

The following information was extracted from the included clinical trials: (1) Type of injury, study design, time to follow-up, when the study was performed (years); (2) Number of participants, mean age and distribution of gender; (3) treatment, drop-out rate, side-effects; (5) type of comparator, drop-out rate, side-effects (4) outcome and its’ measures; (5) risk of bias.

#### Data collection process

Data was extracted from each included study and inserted into a table by one reviewer. A second reviewer audited the data extraction. Any disagreements were resolved by discussion. Functional outcome was reported by validated assessment instruments for wrist injuries, e.g. Disabilities of the Arm, Shoulder, and Hand (DASH) [13] questionnaire and The Patient-rated wrist evaluation (PRWE) [14] and grip strength was presented as kilograms or percentage of the contralateral side. Quality of life was presented by validated quality of life instruments, such as EuroQoL 5 Dimensions (EQ-5D) [15], 36-item short form (SF-36) [16], World Health Organization Quality of Life (WHOQoL) [17] or 15-Dimensional (15-D) [18]. Minimal clinically important differences (MCID) scores for different outcomes (Table 1) were used to reflect the clinical importance of the measured differences between the treatments. Complications were defined as major if they demanded surgical treatment or produced permanent serious disability. All other complications were defined as minor. Each complication was presumed to occur in one individual, even if some patients inevitably may have been affected by two or more complications, thus overestimating the number of patients with complications. Any statistically significant difference in complications was considered clinically important.

**Table 1:** Outcome measurements used in this meta-analysis regarding fractures of the distal radius, with corresponding minimal clinically important differences (MCID).

Outcomes	MCID	References
DASH	13 Points	[19–22]
EQ-5D	0.074 Points	[23, 24]
Grip strength as kilograms or percentage of the uninjured side	6.5 kilo (19,5%)	[25]

#### Statistical methods

The software Comprehensive Meta Analysis Version 3 was used for the meta-analyses. Random effects models were applied, due to the substantial heterogeneity that was expected regarding populations, interventions, comparators and outcome measures across studies. The principal summary measure was mean difference (MD) for the final follow-up assessment. Standardized mean difference (SMD, Cohens’d), based on the groups’ sample sizes, means and standard deviations, were presented when different assessment instruments were used to measure an outcome. When only one RCT contributed to the results, controlled non-randomized trials and/or registry studies (referred to as cohorts) were used to illustrate the differences between treatment options. For analysis of complications, meta-analyses were performed through calculation of Risk Difference (RD), presented as percentages.

Due to too many comparisons and due to small numbers of patients in each comparison, all results from treatment with K-wires/pinning, nailing and external fixation were pooled into one large group–percutaneous methods. We aimed at presenting follow-up for short- and long term outcome but due to too few publications and a diversity in time horizons of follow-ups, we chose to present results only for the one-year follow-up. The results of studies with interventions or comparators deemed to be too heterogeneous were not synthesized in meta-analyses. Inconsistencies and heterogeneity disclosed by the meta-analyses were considered when the certainty of evidence across studies was assessed.

#### Assessing certainty of evidence across studies using GRADE

The international system GRADE [26] was used to assess the certainty of evidence for efficacy and complications across studies according to the following four levels:

High (⊕⊕⊕⊕)–We are very confident that the true effect lies close to that of the estimate of the effect.
Moderate (⊕⊕⊕❍)–We are moderately confident in the effect estimate: the true effect is likely to be close to the estimate of the effect, but there is a possibility that it is substantially different.
Low (⊕⊕❍❍)–Our confidence in the effect estimate is limited: the true effect may be substantially different from the estimate of the effect.
Very low (⊕❍❍❍)–We have very little confidence in the effect estimate: the true effect is likely to be substantially different from the estimate of the effect.

According to the GRADE system, evidence based on RCTs is initially assessed as high certainty, but can be downgraded for reasons such as risk of bias, inconsistency, indirectness, imprecision and publication bias. The rating of certainty of evidence was guided by the available GRADE literature, and decided through consensus among the authors. The process was audited by an internal group for quality control of the Swedish Agency for Health Technology Assessment and Assessment of Social Services as well as an external council of medical experts.

### Cost analysis

Intervention costs for the most commonly used different treatment methods for radius fracture care in a Swedish setting were estimated using a bottom up approach [27]. The necessary resources for each treatment were first identified and then valued using resource unit costs. Resource utilization was calculated for three different operative treatments (plate fixation, pins, external fixation) as well as non-operative treatment with a plaster cast.

The cost analysis included the cost for staff, operating theatre rental costs (including overhead costs), costs for medical technical equipment, costs for orthopaedic implants, and costs for consumables, S5 Appendix. Calculations were performed only for costs related to the primary treatment opportunity in primary or inpatient care, i.e. no attention was given to for example out-patient clinic follow-ups, or societal costs. Short- or long-term complications were not included in the analysis due to lack of published data. All resources needed for each treatment method were identified by expert senior scientists in the research group.

Time in operating theatre for each type of surgical intervention was estimated from previous publications [28–31]. Time consumption for pre- and post-operative care, hospital inpatient duration and utilization of staff involved in different stages of the different treatments, was calculated based on reported data from three large Swedish general hospitals’ computerized operation planning systems (The Sahlgrenska University hospital, Gothenburg, Skåne University hospital, Malmö, and the hospital Södersjukhuset, Stockholm). Data regarding non-surgical interventions was estimated based on clinical experience of authors CE, CMN and POJ. The time for pre-and post-operative care in the perioperative intensive care ward was estimated to be equally long for all surgical treatments, S5 Appendix.

The material costs (consumables and implants) were estimated by collecting data from the economic departments of three general hospitals in Sweden (The Sahlgrenska University hospital, Gothenburg, Skåne University hospital, Malmö, and the hospital Södersjukhuset, Stockholm). The cost for material was valued using a conservative approach by always using the product with the lowest procured price. The units and unit costs derived from these sources are presented in S5 Appendix.

### Registry analysis

All Swedish health-care givers provide mandatory registration of all out- and in-patient care given in Sweden. Registered data is collected in the Swedish National Patient Registry kept by the National Board of Health and Welfare (www.socialstyrelsen.se). For the purpose of this study, data was ordered for the period 2005–2013 with a selection of all individuals above the age of 50 with a reported International Classification of Disease edition 10 (ICD-10) code for a distal radius fracture. Individuals were counted only once to avoid overestimation of incidences. If a Nordic code for surgical procedures (NOMESCO) code for surgical intervention of radial fractures occurred in the registry within 30 days of the date of a distal radius fracture code, the fracture was considered to have been treated surgically. If no surgical code appeared in the registry as described previously, the fracture was considered to have been treated non-operatively. Incidences were calculated as the number of events divided by the size of the target population according to Statistics Sweden (www.scb.se). The data was connected to the Swedish Causes of Death Registry (http://www.socialstyrelsen.se/register/dodsorsaksregistret) to calculate the risk of death after distal radius fracture injury. All handling of data and calculations of registry data were performed in SPSS (version 23; IBM, NY, USA). All findings were compared to previously published epidemiological literature to detect any suspicion of erroneous registry data received from the registry holder Socialstyrelsen (www.socialstyrelsen.se).

## Results

### Systematic review and meta-analysis

#### Eligible studies

**Fig 1:**
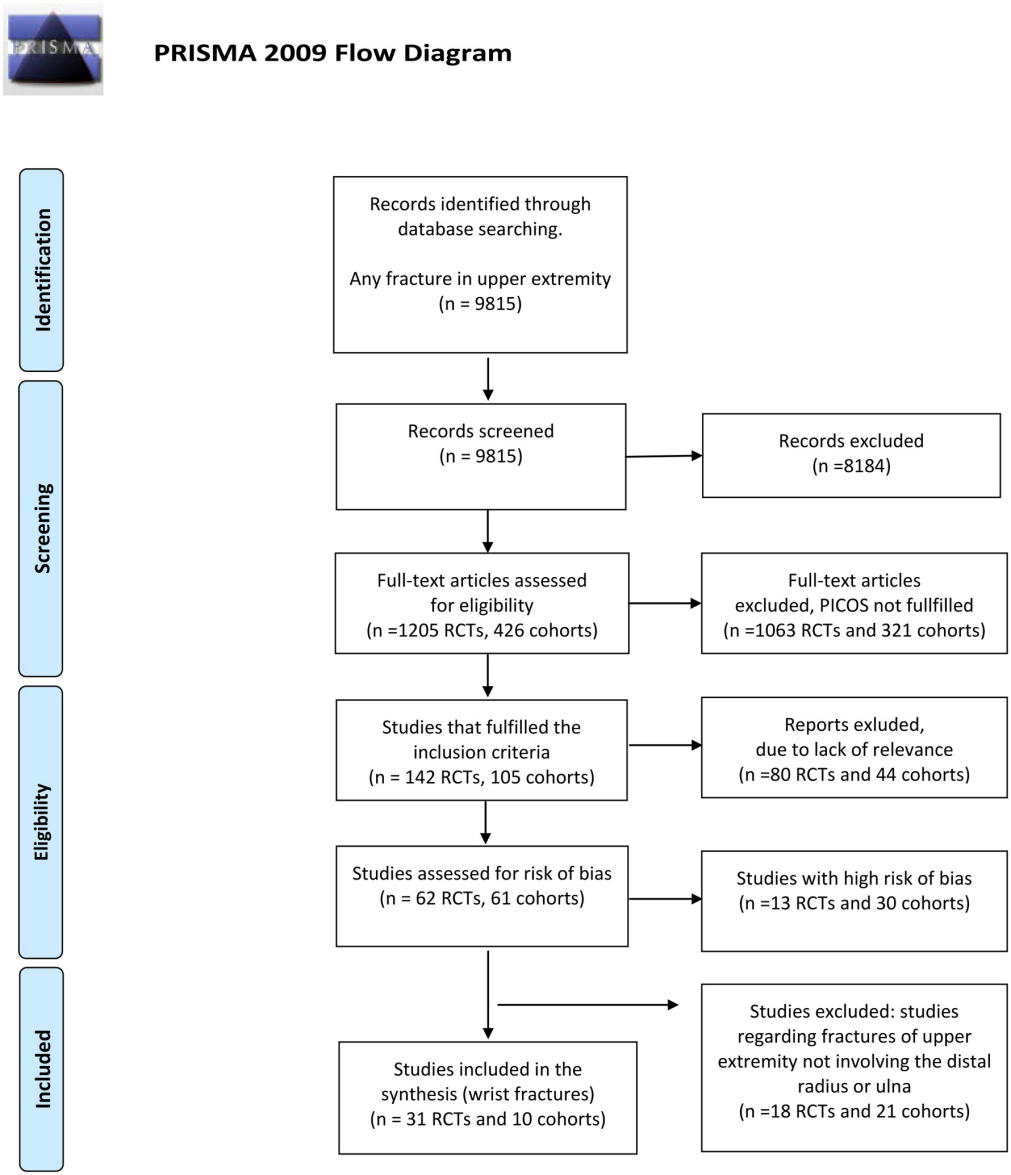
PRISMA flow-chart. *From*: Moher D, Liberati A, Tetzlaff J, Altman DG, The PRISMA Group (2009). *P*referred *R*eporting *I*terns for Systematic Reviews and *M*eta- *A*nalyses: The PRISMA Statement. PLoS Med 6(7): e1000097. doi: 10.1371/journal.pmed.1000097 **For more information, visit** www.prisma-statement.org.

The search of comparative studies regarding fracture treatment in the upper extremity (radius, ulna and humerus) yielded a total of 9815 citations: after review of the abstracts, 8184 were discarded. The full text of a total of 1 205 RCTs and 426 cohort studies citations was examined: 1063 RCTs and 321 cohort studies were excluded as irrelevant, leaving 142 RCTs and 105 cohorts to be evaluated for relevance. Subsequently, 80 RCTs and 44 cohort studies were excluded as not relevant, leaving 62 relevant RCTs and 61 relevant cohort studies for assessment of risk of bias. Of these, 13 RCTs and 30 cohorts were excluded due to high risk of bias, leaving 49 RCTs and 31 cohort studies with low/moderate risk of bias (see S2 Appendix for included studies and S3 Appendix for excluded studies). Studies investigating fractures in the humerus were finally discarded leaving 31 RCTs and 10 cohorts analysing radius and/or ulna fractures for analysis (Fig 1).

The search for health economic evaluations of fracture treatment in the upper extremity yielded a total of 569 potentially relevant citations. Of these, 118 were read in full text and five of these were judged to be relevant [28, 32–35]. Of the five relevant studies, two only focused on resource use or costs [28, 34], one only focused on QALYs [36] and two were full cost-utility analyses (CUA) [33, 35] (see S2 Appendix for included studies and S3 Appendix for excluded studies). Of the two CUA, only one was assessed to be of at least moderate quality [35].

#### Risk of bias in individual studies

All included studies were judged to have moderate or low risk of bias based on a methodological assessment performed by authors CE, CMN, LZ, PN, POJ, and co-worker LEO (mentioned in the Acknowledgement section). All included studies and their risk of bias are presented in S6 Appendix. Data was extracted from studies with a mean age of study populations meeting the inclusion criteria of this meta-analysis. However, some study populations consisted in *only* elderly patients whereas other studies included all ages but with a mean age of study participants matching the inclusion criteria for this meta-analysis (S6 Appendix), which may have introduced bias in our study. Fracture severity, and the description thereof, differed between study populations. One study comparing surgical treatment with non-operative treatment included mildly displaced fractures [37] whereas other studies included severely displaced fractures in a similar comparison [38, 39]. One study was excluded from analysis of functional outcome due to poor validity of the measuring instrument [40]. A large number of cross-overs from non-operative to operative treatment in one study [41] put the analysis of non-operative versus operative treatment at risk of bias.

### Comparison of non-surgical and surgical treatment

#### Study characteristics

Ten trials, eight RCTs and two Non-R studies were included that compared non-surgical and surgical treatment of distal radius fractures in the elderly. In total 730 participants were included of which nine out of ten subjects were women. Of these studies, eight were conducted in Europe, one study in Asia and one in the USA. The treatment methods under analysis were surgical treatment with plate fixation and/or percutaneous fixation (external fixation and/or pinning) as compared to non-surgical treatment (plaster cast). Fractures included were in a majority of cases moderately displaced.

#### Plate fixation versus plaster cast for treatment of distal radius fractures

**Fig 2:**
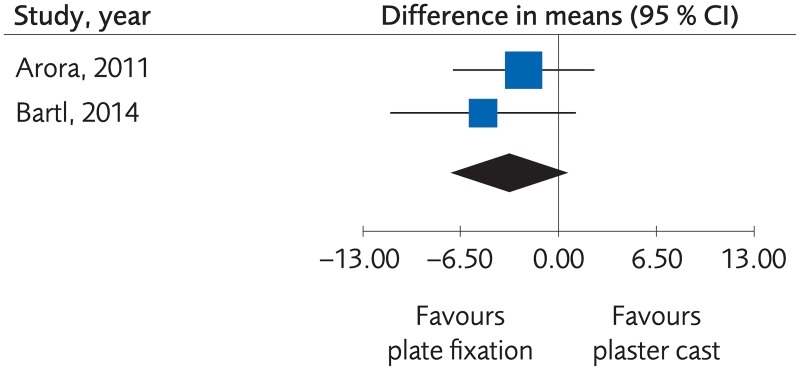
Meta-analysis of randomized controlled trials comparing plate fixation with plaster cast: Results of functional outcome, in elderly patients with distal radius fractures at one-year follow-up.

Two RCTs and one Non-R study compared plate fixation with plaster cast for the functional outcome. There was no clinically important difference at one-year follow-up, (mean difference [MD] -3.29; 95% CI: -7.03 to 0.44, Fig 2). With the low number of trials, the certainty of evidence for the effect on function was rated as moderate (Table 2). There was not enough data for determination of differences regarding grip strength, quality of life or complications.

**Table 2:** Certainty of evidence for surgical versus non-surgical treatment options of distal radius fractures in the elderly, N/A = not applicable.

Intervention	Outcome (measure)	Comparator (C)	N_RCT+Cohort_/Trials [Reference(s)]	Results	Certainty of evidence (GRADE)	Comment
**Surgical v.s non-surgical methods- distal radius fractures**						
Plate fixation	Function	Plaster cast	222+90/ 2 RCTs and 1 cohort/[37, 41], [42]	No clinically important difference MD -3.29 (-7.03; 0.44)	(⊕⊕⊕❍)	-1 risk of bias/indirectness
Plate fixation	Grip strength	Plaster cast	73+90/ 1 RCTs and 1 cohort/[37, 42]	N/A	(⊕❍❍❍)	-2 risk of bias -1 indirectness
Plate fixation	Quality of life	Plaster cast	149/1 RCTs/[41]	N/A	(⊕❍❍❍)	Single study
Plate fixation	Complications	Plaster cast	73+90/1 RCTs and 1 cohort/[37, 42]	N/A	(⊕❍❍❍)	-1 risk of bias -2 indirectness
External fixation/pinning	Function	Plaster cast	205+136/4 RCTs and 2 cohorts/[38, 43–45],[42, 46]	No clinically important difference SMD -0.10 (-0.39; 0.20)	(⊕⊕⊕❍)	-1 indirectness
External fixation/pinning	Grip strength	Plaster cast	312+90/6 RCTs and 1 cohort/[38, 39, 42–45, 47]	No clinically important difference SMD -0.08 (-0.20; 0.35)	(⊕⊕⊕❍)	-1 indirectness
External fixation/pinning	Quality of life	Plaster cast	114/2 RCTs/[43, 45]	Percutaneous methods appear to be as good or better than plaster cast SMD -0.35 (-0.72; 0.02)	(⊕⊕❍❍)	-1 risk of bias -1 indirectness
External fixation/pinning	Complications	Plaster cast	528/ 6 RCTs/[38, 39, 43–45, 47]	Plaster cast appears to give less *minor* complications than percutaneous methods RD 0.13 (0.03; 220)	(⊕⊕❍❍)	-1 risk of bias -1 indirectness
				*Major* complications- N/A	(⊕❍❍❍)	-1 risk of bias -1 indirectness -1 precision
**Surgical v.s non-surgical treatment of distal ulna fractures during concomitant volar plating of the radius**						
Plate fixation of ulna	Function	No fixation of ulna	61/ 1 cohort/[48]	N/A	(⊕❍❍❍)	Single study
Plate fixation of ulna	Grip strength	No fixation of ulna	61/ 1 cohort/[48]	N/A	(⊕❍❍❍)	Single study
Plate fixation of ulna	Quality of life	No fixation of ulna	0/0/-	N/A	(⊕❍❍❍)	No study
Plate fixation of ulna	Complications	No fixation of ulna	61/ 1 cohort/[48]	N/A	(⊕❍❍❍)	Single study

#### Percutaneous surgery versus plaster cast for treatment of distal radius fractures

**Fig 3:**
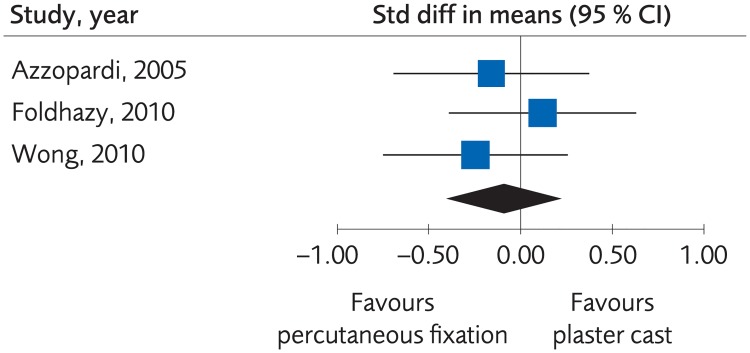
Meta-analysis of randomized controlled trials comparing percutaneous fixation methods with plaster cast: Results of functional outcome, in elderly patients with distal radius fractures at one-year follow-up.

Four RCTs and two Non-R studies compared functional outcome for treatment with percutaneous fixation methods and plaster cast. There were no clinically important differences regarding functional outcome, (standardized mean difference [SMD] -0.10; 95% CI: -0.39 to 0.20, Fig 3). The certainty of evidence was rated as moderate due to the limited number of participants (Table 2).

**Fig 4:**
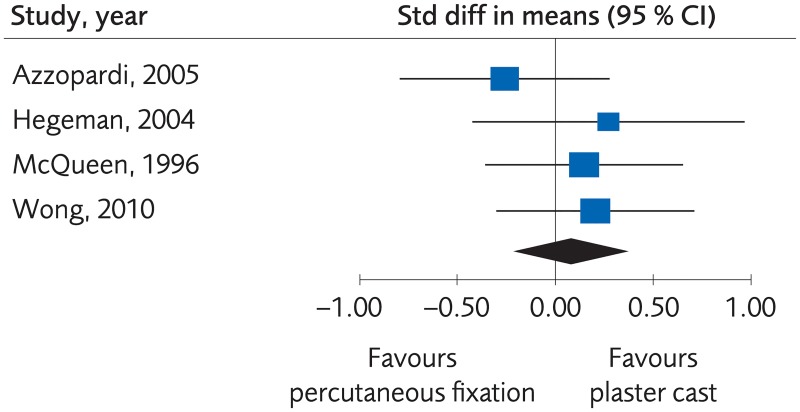
Meta-analysis of randomized controlled trials comparing percutaneous fixation methods with plaster cast: Results of grip strength, in elderly patients with distal radius fractures at one-year follow-up.

Six RCTs and one Non-R study compared the grip strength after treatment with percutaneous fixation methods and plaster cast. There were no clinically important differences regarding grip strength, (SMD 0.08; 95% CI: -0.20 to 0.35, Fig 4), and the certainty of evidence was rated as moderate due to a limited number of participants (Table 2).

**Fig 5:**
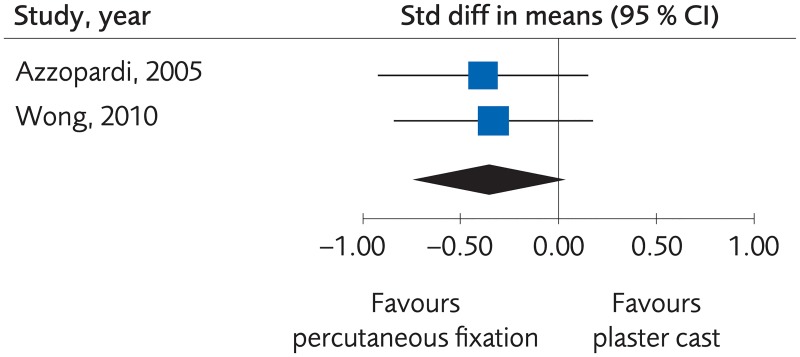
Meta-analysis of randomized controlled trials comparing percutaneous fixation methods with plaster cast: Results of quality of life, in elderly patients with distal radius fractures at one-year follow-up.

Two RCTs compared the quality of life after treatment with percutaneous fixation methods and plaster cast, which resulted in an SMD of -0.35; 95% CI: -0.72 to 0.02, Fig 5. The results suggest that percutaneous treatment is equal or better than non-surgical treatment, but the certainty of evidence was rated as low.

**Fig 6:**
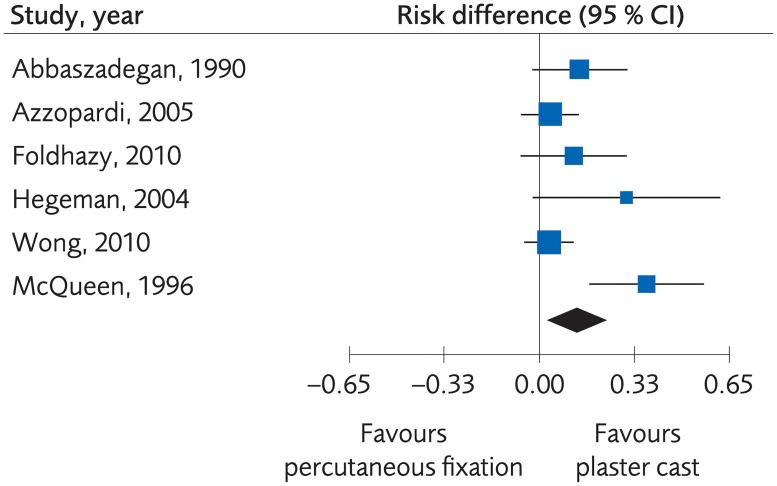
Meta-analysis of randomized controlled trials comparing percutaneous fixation methods with plaster cast: Results of minor complications, in elderly patients with distal radius fractures at one-year follow-up.

Six RCTs reported minor complications. There were fewer complications in the non-surgical treatment group, (Risk difference [RD), 0.13; 95% CI: 0.03 to 0.22, Fig 6. The certainty of evidence was rated as low due to risk of bias and few events (Table 2). The numbers of events were too small to determine differences in major complications.

#### Treatment of distal ulna fractures concomitant to a radial fracture

No studies fulfilling our search strategy were available presenting data on isolated ulna fractures. Only one Non-R study with 61 participants investigated distal ulna fractures concomitant to a radial fracture undergoing plate fixation. Plate fixation of the distal ulna fracture was compared to treatment without operative fixation of the ulnar fracture. There was not enough data to evaluate function, grip strength, quality of life or complications when non-operative treatment was compared to surgical treatment in these patients. Certainty of evidence was rated as very low (Table 2).

### Comparison of different options for surgical treatment of distal radius fractures

#### Study characteristics

Fifteen trials, nine RCTs (1 033 participants) and five Non-R studies and one register study (37 071 participants) were included that compared different surgical treatment options in elderly with distal radius fractures. The subjects were predominantly female. Of these studies, twelve were conducted in Europe and three in Asia. The treatment methods under analysis were operative treatment with different types of plate fixation, plate fixation through different surgical approaches, different percutaneous methods, and plate fixation as compared to percutaneous methods (external fixation and/or pinning).

#### Comparison of different plating techniques

When different types of plate fixations were compared, there was not enough data to present results regarding differences in function, grip strength, quality of life nor complications. The same was true for comparisons of different surgical approaches for plating procedures. The certainty of evidence was rated as very low (Table 3).

**Table 3:** Certainty of evidence for surgical treatment options of distal radius fractures in the elderly, N/A = not applicable.

Intervention	Outcome (measure)	Comparator (C)	N_RCT+Cohort_/Trials [Reference(s)]	Results	Certainty of evidence (GRADE)	Comment
**Surgical v.s surgical methods- distal radius fractures**						
Different types of plate fixations	Function	Different types of plate fixations	42+151/1 RCT and 2 cohorts/ [49–51]	-	(⊕❍❍❍)	N/A
Different types of plate fixations	Grip strength	Different types of plate fixations	42+106/ 1 RCT and 1 cohort/[49, 51]	-	(⊕❍❍❍)	N/A
Different types of plate fixations	Quality of life	Different types of plate fixations	**-**	-	(⊕❍❍❍)	No study
Different types of plate fixations	Complications	Different types of plate fixations	42+151/1 RCT and 2 cohorts/ [52, 53]	-	(⊕❍❍❍)	N/A
Different surgical approaches for plating procedures	Function	Different surgical approaches for plating procedures	38/ 1 RCT/ [54]	-	(⊕❍❍❍)	N/A
Different surgical approaches for plating procedures	Grip strength	Different surgical approaches for plating procedures	96/ 2 RCTs/[54, 55]	-	(⊕❍❍❍)	N/A
Different surgical approaches for plating procedures	Quality of life	Different surgical approaches for plating procedures	**-**	-	(⊕❍❍❍)	No study
Different surgical approaches for plating procedures	Complications	Different surgical approaches for plating procedures	240/ 2 cohorts/[52, 53]	-	(⊕❍❍❍)	N/A
Different percutaneous methods: Bridging external fixation	Function	Different percutaneous methods: Non-bridging external fixation	38/1 RCT/[54]	**-**	(⊕❍❍❍)	Single study
Different percutaneous methods: Bridging external fixation	Grip strength	Different percutaneous methods: Non-bridging external fixation	96/2 RCTs/[54, 55]	No clinically important difference	(⊕⊕❍❍)	-1 risk of bias-1 indirectness
Different percutaneous methods: Bridging external fixation	Quality of life	Different percutaneous methods: Non-bridging external fixation	38/1 RCTs/[54]	**-**	(⊕❍❍❍)	Single study
Different percutaneous methods	Complications	Different percutaneous methods	177/3 RCTs/[40, 54, 55]	-	(⊕❍❍❍)	-1 indirectness-2 inconsistency
Plate fixation	Function	Percutaneous fixation	812+30/5 RCTs and 1 cohort/[50, 56–60]	No clinically important difference SMD -0.07 (-0.21 to 0.07)	(⊕⊕⊕❍)	-1 risk of bias
Plate fixation	Grip strength	Percutaneous fixation	397+62/ 4 RCTs and 1 cohort/[57–61]	No clinically important difference MD -3.47 (-11.21 to 4.28)	(⊕⊕⊕❍)	-1 risk of bias/ indirectness
Plate fixation	Quality of life	Percutaneous fixation	549/2 RCTs/[56, 60]	No clinically important difference MD: -0.02 (-0.06 to 0.02)	(⊕⊕❍❍)	-1 risk of bias-1 indirectness
Plate fixation	Complications	Percutaneous fixation	812+ 36 648/5 RCTs and 2 cohorts/[29, 50, 56–60]	No clinically important difference- minor complications RD: -0.01 (-0.07 to 0.05) Percutaneous fixation appears to give less *major* complications than plate fixation RD^RCT+Cohort^: 0.02 (0.02 to 0.03)	(⊕⊕⊕❍)	-1 risk of bias

#### Comparison of different percutaneous techniques

Analysis of differences in function, quality of life and complications regarding different percutaneous methods failed due to too few observations. The certainty of evidence was rated as very low. Two RCTs compared bridging and non-bridging external fixation in elderly patients with distal radius fractures. No clinically important differences were seen. The certainty of evidence was rated as low, due to the number of participants.

#### Plating versus percutaneous techniques

**Fig 7:**
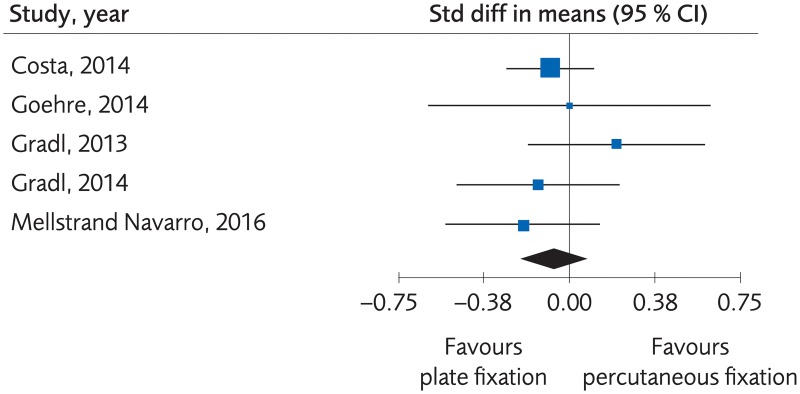
Meta-analysis of randomized controlled trials comparing plate fixation with percutaneous fixation methods: Results of functional outcome, in elderly patients with distal radius fractures at one-year follow-up.

**Fig 8:**
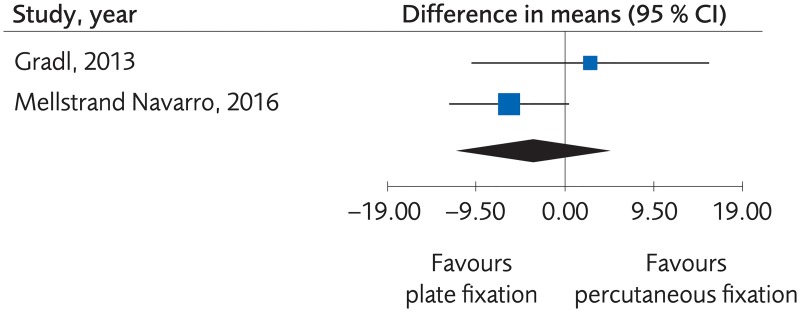
Meta-analysis of randomized controlled trials comparing plate fixation with percutaneous fixation methods: Results of grip strength, in elderly patients with distal radius fractures at one-year follow-up.

**Fig 9:**
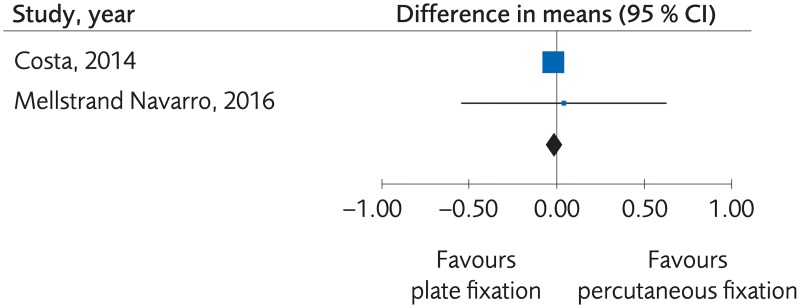
Meta-analysis of randomized controlled trials comparing plate fixation with percutaneous fixation methods: Results of quality of life, in elderly patients with distal radius fractures at one-year follow-up.

**Fig 10:**
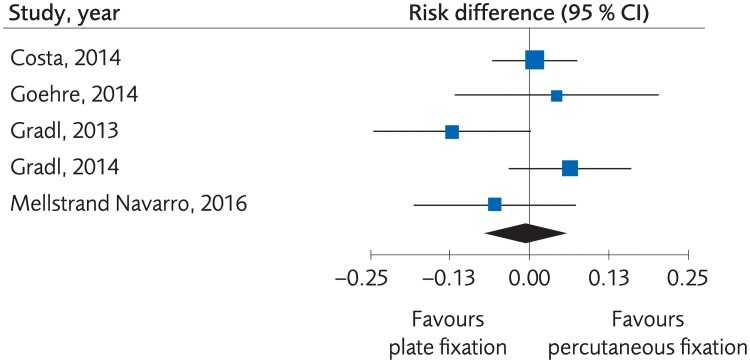
Meta-analysis of randomized controlled trials comparing plate fixation with percutaneous fixation methods: Results of minor complications, in elderly patients with distal radius fractures at one-year follow-up.

**Fig 11:**
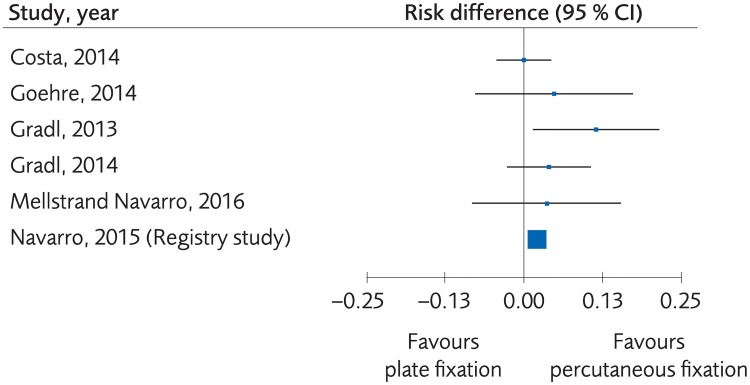
Meta-analysis of randomized controlled trials and one registry study comparing plate fixation with percutaneous fixation methods: Results of major complications, in elderly patients with distal radius fractures at one-year follow-up.

Five RCTs and one Non-R study investigated plate fixation compared to percutaneous fixation. No clinically important differences were seen regarding clinical function, (SMD -0.07, 95% CI: -0.21 to 0.07, Fig 7) or grip strength, (MD -3.47, 95% CI: -11.21 to 4.28, Fig 8), certainty of evidence was rated as moderate. No clinically significant differences were seen regarding quality of life, (MD -0.02, 95% CI: -0.06 to 0.02, Fig 9) certainty of evidence was rated as low. There were no differences for minor complications, (RD -0.01, 95% CI: -0.07 to 0.05, Fig 10) whereas for major complications there was an advantage for the percutaneous group, (RD 0.02, 95% CI: 0.02 to 0.03, Fig 11). Certainty of evidence was rated as moderate.

### Comparison of addition of bone substitute

#### Study characteristics

Seven RCTs (673 participants) were included that compared different aspects of addition of bone substitute in elderly with distal radius fractures. The subjects were predominantly female. Out of these studies, five were conducted in Europe, one in Asia and one in the USA.

#### Plating with or without bone substitute

When use of bone substitute as addition to plate fixations was analysed, there was not enough data to present results regarding differences in clinical function, grip strength, quality of life nor complications. The certainty of evidence was rated as very low (Table 4).

**Table 4:** Certainty of evidence for bone substitute as addition to plate or percutaneous fixation as treatment options of distal radius fractures in the elderly.

Intervention	Outcome (measure)	Comparator (C)	N_RCT+Cohort_/Trials [Reference(s)]	Results	Certainty of evidence (GRADE)	Comment
**Surgical v.s surgical methods- distal radius fractures**						
Bone substitute as addition to plate fixation	Function	**No** bone substitute as addition to plate fixation	80/2 RCTs / [62, 63]	-	(⊕❍❍❍)	-1 risk of bias -2 indirectness
Bone substitute as addition to plate fixation	Grip strength	**No** bone substitute as addition to plate fixation	80/2 RCTs /[62, 63]	-	(⊕❍❍❍)	-1 risk of bias -2 indirectness
Bone substitute as addition to plate fixation	Quality of life	**No** bone substitute as addition to plate fixation	**-**	-	(⊕❍❍❍)	No study
Bone substitute as addition to plate fixation	Complications	**No** bone substitute as addition to plate fixation	80/2 RCTs /[62, 63]	-	(⊕❍❍❍)	-1 risk of bias -2 indirectness
Bone substitute to stabilize plaster alone, or percutaneous pinning	Function	**No** bone substitute to stabilize plaster alone, or percutaneous pinning	485/ 3 RCTs/[64–66]	Bone substitute was as good as or better than without bone substitute SMD -0.44 (-0.97 to 0.10)	(⊕⊕❍❍)	-1 imprecision -1 inconsistency
Bone substitute to stabilize plaster alone, or percutaneous pinning	Grip strength	**No** bone substitute to stabilize plaster alone, or percutaneous pinning	593/ 5 RCTs/ [39, 64–67]	No clinically important difference MD: -10.42 (-17.91 to -2.94)	(⊕⊕❍❍)	-1 risk of bias -1 inconsistency
Bone substitute to stabilize plaster alone, or percutaneous pinning	Quality of life	**No** bone substitute to stabilize plaster alone, or percutaneous pinning	323/ 1 RCT/[64]	-	(⊕❍❍❍)	Single study
Bone substitute to stabilize plaster alone, or percutaneous fixation	Complications	**No** bone substitute to stabilize plaster alone, or percutaneous pinning	593/ 5 RCTs/ [39, 64–67]533/ 4 RCTs/[64–67]	No clinically important difference-*minor* complications RD: -0.02 (-0.16 to 0.13)	(⊕⊕❍❍)	-1 risk of bias -1 indirectness
				No bone substitute was as good as or better than bone substitute for percutaneous fixation-*major complications* RD: 0.03 (-0.002 to 0.05)	(⊕⊕❍❍)	-1 risk of bias -1 indirectness

#### Percutaneous or plaster treatment with or without bone substitute

**Fig 12:**
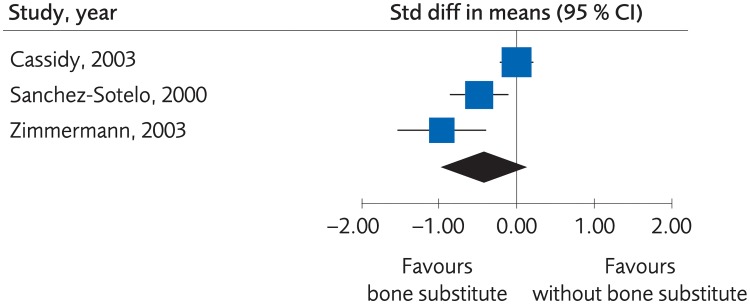
Meta-analysis of randomized controlled trials comparing percutaneous or plaster treatment with or without bone substitute: Results of functional outcome, in elderly patients with distal radius fractures at one-year follow-up.

**Fig 13:**
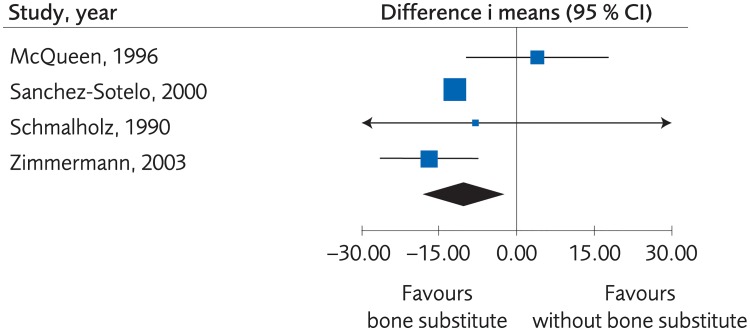
Meta-analysis of randomized controlled trials comparing percutaneous or plaster treatment with or without bone substitute: Results of grip strength, in elderly patients with distal radius fractures at one-year follow-up.

**Fig 14:**
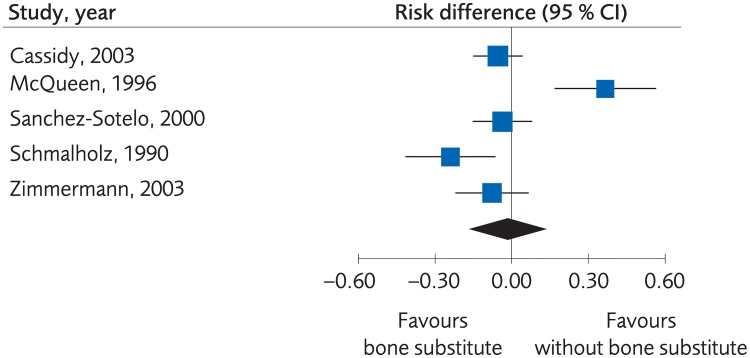
Meta-analysis of randomized controlled trials comparing percutaneous or plaster treatment with or without bone substitute: Results of minor complications, in elderly patients with distal radius fractures at one-year follow-up.

**Fig 15:**
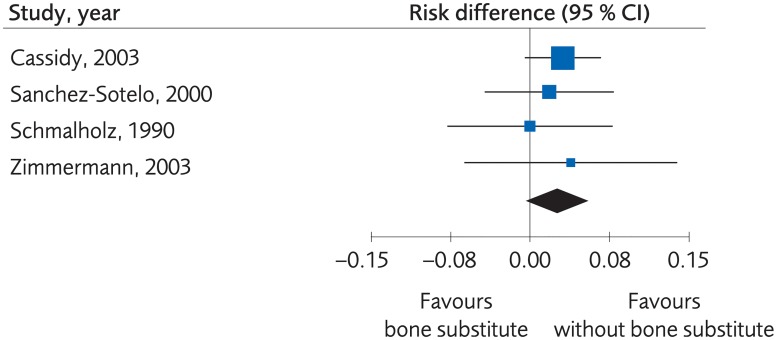
Meta-analysis of randomized controlled trials comparing percutaneous or plaster treatment with or without bone substitute: Results of major complications, in elderly patients with distal radius fractures at one-year follow-up.

When the fracture was stabilized by plaster alone, or by percutaneous pinning, bone substitute proved to yield equal or better clinical results than no bone substitute, (SMD -0.44, -0.97 to 0.10, Fig 12). However, the evidence was rated as low due to risk of bias and lack of consistency between the studies. Moreover, there were no clinically important differences regarding grip strength, (MD -10.42, -17.91 to -2.94, Fig 13), and the certainty of evidence was rated as low. There was not enough data to present results regarding differences in quality of life, certainty of evidence was rated as very low. There were no differences between methods regarding minor complications, (RD 0.03, 95% CI: -0.002 to 0.05, Fig 14) whereas no bone substitute was equally good or better regarding major complications, (RD 0.03, 95% CI:0.002 to 0.05, Fig 15), with certainty of evidence rated as low.

### Comparison of different options for non-surgical treatment

#### Study characteristics

Two RCTs (163 participants) and one Non-R study (83 participants) were included that compared different non-surgical treatment options in elderly with distal radius fractures. The subjects were predominantly female. All three studies were conducted in Europe.

The non-operative treatment regime comparisons were time in plaster immobilization and efficacy of closed reduction respectively.

The two RCTs compared 3 weeks of fixation in a plaster cast to 5 weeks of fixation. There was no clinically important difference between treatment options regarding grip strength. The certainty of evidence was rated as low, due to the low number of participants and the risk of bias in the studies. There was not enough data to present differences regarding function, quality of life nor complications.

Closed reduction was compared to plaster treatment in situ in one Non-R study. There were too few observations to present any differences regarding function, grip strength, quality of life or complications. The certainty of evidence was rated as very low.

### Health economy evaluation

#### Health economy literature review

Two economic evaluations treating distal radius fracture care were identified [35, 68]. However, only one was assessed to have at least moderate quality. Tubeuf and colleagues [35] evaluated operative treatment with plate fixation compared to operative treatment with percutaneous fixation (Kirschner wires) over a time perspective of 12 months. Results for patients over 50 years were presented in a subgroup analysis. From a NHS (the British National Health Service) and Personal Social Service perspective, the analysis resulted in a gain of 0.014 QALYs (0.002–0.025) and a cost per QALY of USD 81997 (GBP 54218 year 2012). Using a societal perspective including production losses, the cost per QALY decreased to USD 53421 (GBP 35323 year 2012) per QALY.

#### Cost analysis

Interventional costs for treatment of distal radius fractures in a Swedish setting, is shown in Table 5. The intervention cost ranged from USD 137 for treatment with a cast to USD 1 698 for surgical treatment with a plate fixation, which was the most expensive treatment method. The cost for surgical treatment with plate fixation was higher than that with external fixation and pins. This higher cost can be explained by both higher implant costs and longer time in operating theatre.

**Table 5:** Intervention costs per treatment of distal radius fracture in a Swedish setting. Estimation performed within the context of a health economy analysis performed by the SBU.

	Intervention costs (USD 2016)			
Resources	Plaster cast	Plate fixation	Pins	External fixation
Material	6	233	26	142
Time in the operating theatre including fixed equipment + overhead costs		540	445	445
Orthopaedic surgeon	95	214	143	143
Assisting orthopaedic surgeon		83	48	48
Anaesthesist		107	107	107
Anaesthetic nurse		193	159	159
Surgical nurse		193	159	159
Operation assistant		135	111	111
Out-patient clinic nurse	36			
Total	137	1 698	1 198	1 314

USD: United states dollars

### Registry analysis

**Fig 16:**
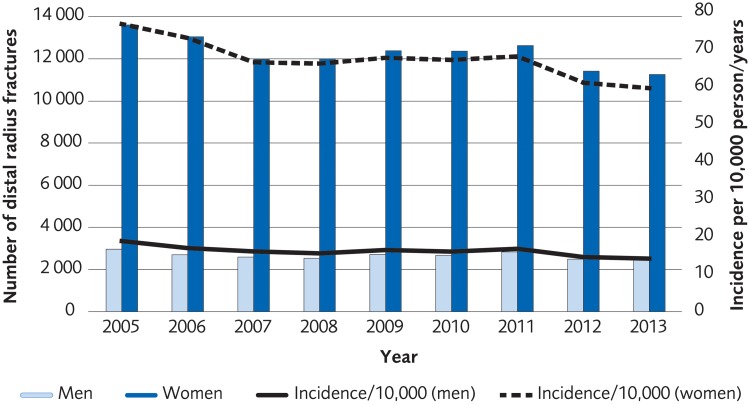
Incidence per 10,000 person-years of distal radius fractures in the Swedish population ≥50 years old, between 2005 and 2013.

**Fig 17:**
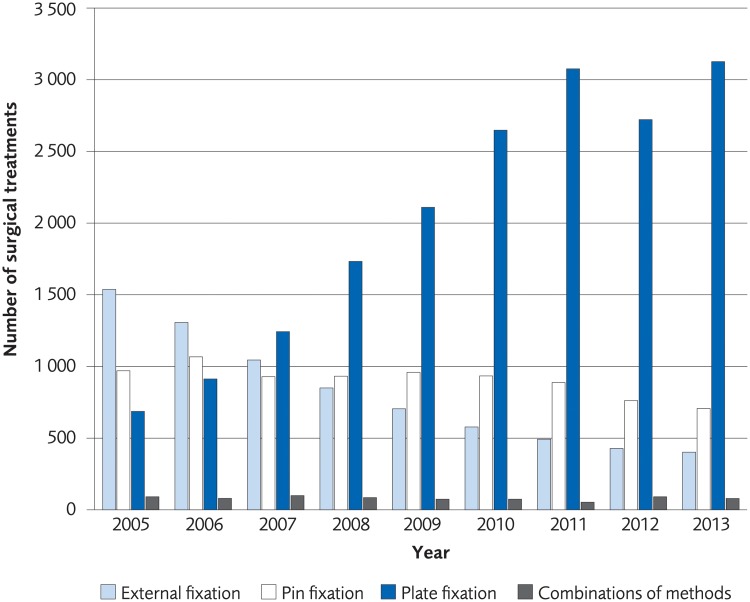
The most common surgical treatments of distal radius fracture in women, ≥50 years old, between 2005–2013 in Sweden.

**Fig 18:**
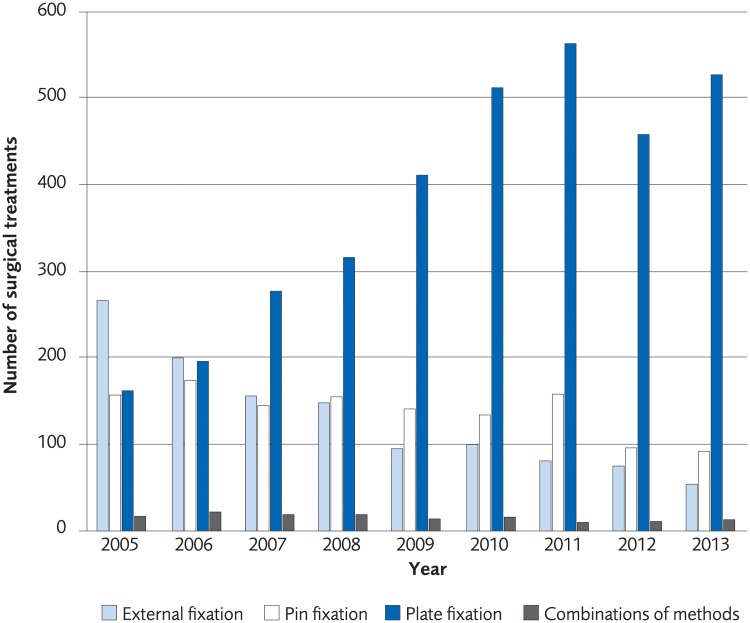
The most common surgical treatments of distal radius fracture in men, ≥50 years old, between 2005–2013 in Sweden.

The analysis was performed on registry data from 110 540 women and 23 913 men over the age of 50 who according to registrations in the Swedish National Patient Registry sustained a distal radius fracture during the period 2005 to 2013. The incidence of a distal radius fracture in patients over 50 years old was 77/10,000-person years among women in 2005 and 63/10,000-person years in 2013. The incidence of a distal radius fracture in men in the same age group was 18/10 000-person years in 2005 and 14/10 000 in 2013 (Fig 16). The proportion of patients undergoing surgical treatment increased by 6.7% for women and 4.2% for men during 2005–2013. The most common method for surgical treatment in 2005 was external fixation, but the use of plate fixation increased during the time under investigation to become the most used method in 2007 through 2013 (Fig 17, for women, Fig 18 for men). Risk of death within 30 days from the distal radius fracture was 0.5%.

## Discussion

The main finding of this systematic review was that there was no difference in clinical outcome between surgical and non-surgical treatment of moderately displaced distal radius fractures when analysing currently available literature on treatment in the elderly. The same was true when comparing different surgical techniques, although plating yielded a higher number of complications. Moreover, the estimation of costs for distal radius fracture treatments showed that plating was more expensive than percutaneous pinning and external fixation. The least expensive treatment option was, not unexpectedly, non-operative treatment. Our analysis of Swedish registry data regarding distal radius fracture incidences and treatment showed that incidence of distal radius fractures tended to decrease 2005–2013, the proportion of surgical treatment of distal radius fractures increased during the same time period, and plating was the most widespread surgical technique used for distal radius fracture fixation in 2013.

A systematic review of literature of outcomes and complications of distal radius fractures in the elderly by Diaz-Garcia supports our findings [69]. The inclusion criteria for their literature search were identical to ours, except that they accepted articles with somewhat smaller study populations in each comparative group. They report small but clinically insignificant differences in clinical outcome between cast immobilization, volar plating, bridging external fixation and non-bridging external fixation. Similarly, they also found a higher reoperation rate after plate fixation.

Contrary to our findings, Esposito and colleagues present data with an advantage for patients treated with a plate, as compared to treatment with external fixation [70]. Only RCTs were included in their meta-analysis and the patient populations were generally younger than in our study. Esposito and colleagues present a significant MD of 5.92 in DASH with better results for plate fixation. According to other authors, this is not regarded a clinically important difference [19–22]. Similar to our findings, Esposito and colleagues also present no differences in grip strength between groups. They also present data for range of motion and radiology, which we have not included in our analysis. Esposito found no difference in overall complications between groups, except for infection that was more common in the external fixation group.

Similar to our findings, Chen and colleagues [71] report in their meta-analysis of distal radius fracture treatment in elderly patients that there was no clinically significant difference between surgical treatment and non-surgical treatment as measured by the DASH. However, they report significantly better radiographic findings after surgery than after non-surgical treatment. The clinical value of radiography does not seem to be important since the DASH did not differ between groups. We did not present data on radiologic healing or fracture malunion in our meta-analysis. There are multiple reasons for this decision. Firstly, in accordance with Chen and colleagues, there are many publications supporting that there is no strong association between the radiographic appearance and the clinical results in elderly patients with radial fractures [72–75]. We considered clinical outcome such as DASH and grip-strength as more important clinical outcomes. Secondly, we considered the difficulties in obtaining reliable radiological measurements. There are large differences in quality of postoperative radiographs. Moreover, there is evidence of intra- and inter-observer measurement errors of radiographic analysis [76]. Our choice to exclude radiologic analysis of distal radius fracture outcomes is supported by Costa and colleagues [77], who did not present any radiographic data in their multicentre randomized controlled trial on distal radial fracture treatment.

It could be argued that short term outcome is important to elderly patients. However, there was not sufficient data for analysis of short term result in our meta-analysis of clinical outcome. A faster return to normal may be of great importance for an elderly patient, who might lose living independence if there are disabilities of the wrist during the first months after injury. There are reports on an advantage for surgical treatment with a plate at early follow-ups, but the differences had evened out at the one-year follow-up [78–81]. More research is needed to elucidate the value of early recovery in elderly patients with distal radius fractures.

Our study failed to present support for a value of surgical treatment in elderly patients. However, it is important to note what kind of fracture populations that have been studied in the randomized trials included. In the study of Arora and colleagues [37], there were no clinical differences between the patients treated non-operatively and those treated with a volar plate. The radiographic end-result in the non-surgically treated group was a mean dorsal tilt of 10.4 degrees, i.e., fractures treated non-surgically healed in only a slight displacement from the anatomical position. No preoperative radiology was presented in the study from Arora. Non-operatively treated fractures in the elderly tend to heal in the original position of fracture displacement [73]. Hence, there is reason to believe that the fractures included in Arora’s study were only moderately displaced before treatment was initiated. The results of Arora and colleagues [37] cannot be generalized for a population with more severely displaced fractures. The same is true for the results from Bartl and colleagues 2014 [41]. In their study, 42% of patients in the group allocated to non-operative treatment crossed over to surgical treatment due to fracture displacement. As a result, very little information was added to the knowledge of clinical result after completed non-operative treatment of displaced fractures. In summary, the results from these two studies comparing non-operative and surgical treatment present knowledge on how moderately displaced fractures behave after non-operative treatment. These results may not be applicable to patients with severely displaced fractures. Since we performed our literature search, a new randomized controlled trial has been published comparing operative and non-operative treatment for distal radius fractures in the elderly [7]. Contrary to our findings, they report an advantage with volar plating as compared to non-operative treatment. Elderly populations deserve more investigations in high quality randomized trials [82].

When comparing the results after distal radius treatment we have limited our presentation to one-year follow-up. No long-term data was collected for our analysis. In our clinical experience, there is no reason to suspect that the clinical result would change largely after one year, even if very little data has been published on this matter. However, long term follow-up may be of interest to detect late complications, which are known to occur e g. after volar plating [29, 83–86]. This may also have impact on health-economic analyses, since frequent late reoperations would have an impact on the costs as well as QALYs associated with different treatment methods.

In any orthopaedic practise treating wrist fractures, a common clinical controversy is whether or not to perform surgical fixation of a concomitant ulna fracture. When presenting our findings of the literature search there was a surprising paucity of publications regarding how to treat ulnar fractures. The same knowledge gap exists for most non-operative alternatives for distal radius fractures, e g type of plaster cast, over the elbow fixation or not, orthosis, position in cast, immobilization time etcetera.

Our analysis of Swedish registry data regarding treatment choices of distal radius fractures showed that surgical treatment is increasing, and more specifically, treatment with a plate is increasing. When new medical treatment methods are implemented and used within any health care system there should be a reasonable relationship between costs and effect. The increased investment in more expensive treatments demonstrated in this report cannot be motivated from a health economic perspective with regards to validated outcome measures. It would be of interest to know more about the forces behind the increase in more costly treatment. In the case of distal radius fracture treatment, recent changes of treatment traditions do not appear to be evidence based.

### Limitations

Some limitations of our review should be noted. Firstly, we solely relied on information available in the published reports. Some reports did not, for instance, clearly indicate the severity of the fracture or the quality of the bone. The day the surgery was performed in relation to how many days that had passed since the occasion of the fracture may influence the complexity and outcome of the surgery. This important information was presented only in few studies. The number of individuals included in the analyses at a follow-up was not clear in all studies and in such cases we assumed that all randomized participants were included, which might not always have been the case. Secondly, we only report outcomes at one-year follow-up, due to a low number of included trials that reported shorter or longer follow-up data. Short-term outcomes may differ between treatment options but we have not taken that into consideration in our meta-analysis. Thirdly, and related, we could not properly assess the risk of publication bias. The low number of included trials–in combination with the heterogeneous interventions, comparators, and populations–made statistical tests of publication bias unreliable.

The assessment of risk of bias and the use of GRADE in the present review might be regarded as overly stringent. Exclusion of trials with a high risk of bias reduced the number of included trials. On the other hand, a major advantage of GRADE is that it provides a framework for guidance through the critical components of the assessment and provides an approach to analysis and communication that encourages transparency and an explicit accounting of the judgements involved [26].

Our estimations of costs for different radial fracture treatments did not include sick leave, consumption of pain killers, antibiotics, postoperative out-patient clinic visits and other possible points of expenses. A more thorough health economic evaluation might yield other conclusions. Another limitation is that there was no report on other treatment, such as physiotherapy, in the included studies, which might influence the functional outcomes.

The presentation of epidemiological data from, the Swedish National Patient Registry (NPR) always carries the risk of inconsistent or erroneous data due to missing data or falsely reported registry data. However, there are publications reporting high validity of the NPR [87] and we consider our epidemiological data sufficiently robust to allow publication.

## Conclusions

Despite the limitations and the relatively few studies included in this meta-analysis, our study present data supporting that there is no evidence suggesting added value of surgical treatment in elderly patients with moderately displaced distal radius fractures. In the future, it will be important to focus on methodologically well conducted RCTs investigating functional recovery, complications and quality of life in patients with distal radius fractures and study populations should be clearly described regarding age, sex, bone quality and severity of the fracture. To contribute to higher certainty of evidence, future studies need to have a sufficient number of individuals included, present data on short- and long-term follow-up and use validated measurement instruments.

## Supporting information

S1 Appendix Search strategy. (PDF)

S2 Appendix Included publications. (PDF)

S3 Appendix Excluded publications. (PDF)

S4 Appendix PRISMA check-list. (PDF)

S5 Appendix Cost analysis. (PDF)

S6 Appendix Risk of bias. (PDF)
