# Supplementary material for: Treatment of radius or ulna fractures in the elderly: A systematic review covering effectiveness, safety, economic aspects and current practice
Source: PLoS One. 2019 Mar 28;14(3):e0214362. doi: 10.1371/journal.pone.0214362 (PMC6438530; doi:10.1371/journal.pone.0214362)
Supplement: S1 Appendix — (PDF) [file pone.0214362.s001.pdf]

S1 Appendix.

Search strategy for the purpose of a literature review performed by the Swedish Agency for Health Technology Assessment and Assessment of Social Services

Effectiveness and complications of treatments

PubMed via NLM 01 December 2016

Title: Treatment options for fractures of the upper extremity in the elderly

Search terms		Items found
**Population: Arm fractures**		
1.	"Osteoporotic Fractures"[Mesh] OR "Osteoporosis/surgery"[Mesh] OR "Radius Fractures"[Majr] OR "Colles' Fracture"[Majr] OR "Ulna Fractures"[Majr] OR "Humeral Fractures"[Majr] OR "Shoulder Fractures"[Majr] OR "Wrist Injuries"[Majr] OR humeral[Title] OR distal radial[Title] OR osteoporo*[Title] OR ulna[Title]	49337
2.	(((osteoporo*[Title/Abstract] OR distal radial[Title/Abstract] OR distal radius[Title/Abstract] OR humerus[Title/Abstract] OR humeral[Title/Abstract] OR humerous[Title/Abstract] OR supracondylar[Title/Abstract] OR ulna[Title/Abstract] OR wrist*[Title/Abstract] OR collum chirurg*[Title/Abstract] OR elbow*[Title/Abstract] OR olecranon[Title/Abstract] OR shoulder*[Title/Abstract] OR arm[Title/Abstract] OR upper extremit*[Title/Abstract] OR upper limb*[Title/Abstract] OR forearm*[Title/Abstract] OR fore arm*[Title/Abstract] OR barton*[Title/Abstract] OR colle*[Title/Abstract]) AND fracture*[Title/Abstract]) NOT medline[SB])	7607
3.	1 OR 2	55029
**Interventions**		
4.	"surgery" [Subheading] OR "Orthopedic Fixation Devices"[Majr] OR "Bone Nails"[Majr] OR "Bone Plates"[Majr] OR "Bone Screws"[Majr] OR "Bone Wires"[Majr] OR "Casts, Surgical"[Majr] OR "External Fixators"[Majr] OR "Internal Fixators"[Majr] OR "Splints"[Majr] OR "Suture Anchors"[Majr] OR "Orthotic Devices"[Mesh] OR "Athletic Tape"[Mesh] OR "Braces"[Mesh]OR "Fracture Fixation, Internal"[Majr] OR "Fracture Fixation, Intramedullary"[Majr] OR "Fracture Fixation/methods"[Majr] OR "Fracture Fixation/methods"[Mesh] OR "Arthroplasty/methods"[Mesh] OR plate*[Title] OR nail*[Title] OR fixation[Title]	1928926
5.	(nail*[Title/Abstract] OR plate[Title/Abstract] OR plating[Title/Abstract] OR plates[Title/Abstract] OR pin[Title/Abstract] OR pins[Title/Abstract] OR pinning*[Title/Abstract] OR implant*[Title/Abstract] OR screw*[Title/Abstract] OR wire*[Title/Abstract] OR k-wire*[Title/Abstract] OR kirschner*[Title/Abstract] OR splint*[Title/Abstract] OR tension band*[Title/Abstract] OR orthotic[Title/Abstract] OR orthosis[Title/Abstract] OR tape[Title/Abstract] OR plaster[Title/Abstract] OR brace*[Title/Abstract] OR collar[Title/Abstract] OR slab[Title/Abstract] OR bridging[Title/Abstract] OR non-bridging[Title/Abstract] OR nonbridging[Title/Abstract] OR external fixat*[Title/Abstract] OR internal fixat*[Title/Abstract] OR prosthes*[Title/Abstract] OR cast[Title/Abstract] OR casts[Title/Abstract] OR plaster*[Title/Abstract] OR arthroplast*[Title/Abstract] OR non-surgical treatment*[Title/Abstract] OR nonsurgical treatment*[Title/Abstract] OR conservative treatment*[Title/Abstract]) NOT medline[SB])	106115

6.	4 OR 5	2027904
**Combined sets**		
7.	3 AND 6	18105
**Study types: randomised controlled trials and other trials (filter: PubMed clinical queries, therapy, broad) [1] Added terms for: case-control, registry, cohort**		
8.	((clinical[Title/Abstract] AND trial[Title/Abstract]) OR clinical trials[MeSH Terms] OR clinical trial[Publication Type] OR random*[Title/Abstract] OR random allocation[MeSH Terms] OR therapeutic use[MeSH Subheading]) OR "Comparative Study" [Publication Type] OR "Evaluation Studies" [Publication Type] OR "Cohort Studies"[Mesh] OR "Longitudinal Studies"[Mesh] OR "Prospective Studies"[Mesh] OR "Retrospective Studies"[Mesh] OR "Case-Control Studies"[Mesh] OR cohort*[Title/Abstract] OR longitudinal[Title/Abstract] OR prospective[Title/Abstract] OR retrospective[Title/Abstract] OR observational[Title/Abstract] OR registr*[Title/Abstract] OR case-control*[Title/Abstract]	7374170
**Limits:**		
9.	(animals [MeSH] NOT humans [MeSH])	4230327
10.	Filters: **Publication date from 1990/01/01 to 2016/12/31; English; Danish; Norwegian; Swedish**	
11.	**(7 AND 8 AND 10) NOT 9**	**6307**

The search result, usually found at the end of the documentation, forms the list of abstracts.

[MeSH] = Term from the Medline controlled vocabulary, including terms found below this term in the MeSH hierarchy
[MeSH:NoExp] = Does not include terms found below this term in the MeSH hierarchy
[MAJR] = MeSH Major Topic
[Title/Abstract] = Title or abstract
[Title] = Title
[AU] = Author
[TW] = Text Word
Systematic[SB] = Filter for retrieving systematic reviews
* = Truncation

[1] Haynes RB, McKibbon KA, Wilczynski NL, Walter SD, Werre SR, Hedges Team. Optimal search strategies for retrieving scientifically strong studies of treatment from Medline: analytical survey. BMJ 20 = 614181305;330(7501):1179.

Cochrane Library via Wiley 01 December 2016 (CDSR, DARE & CENTRAL)

Title: Treatment options for fractures of the upper extremity in the elderly

Search terms		Items found
**Population: Arm fractures**		
1.	[mh "osteoporotic fractures"] or [mh osteoporosis/PX,SU] or [mh "radius fractures"] or [mh "colles' fracture"] OR [mh "ulna fractures"] OR [mh "humeral fractures"] OR [mh "shoulder fractures"] OR [mh "wrist injuries"]	941
2.	((osteoporo* or "distal radial" or "distal radius" or humerus or humeral or humerous or supracondylar or ulna or wrist* or "collum chirurgicum" or elbow* or olecranon or shoulder* or arm or “upper extremities” or “upper extremity” or "upper limb" or "upper limbs" or forearm* or "fore arm" or barton* or colle*) near/4 fracture*):ti,ab,kw	3051
3.	1 OR 2	3168
**Population: Age**		
4.	[mh^adult] or [mh aged] or [mh "middle aged"] or [mh "aged, 80 and over"] OR [mh "frail elderly"] or [mh menopause] or [mh "age factors"] or (adult* or old or older):ti or (aging or elderly or elder or aged or "old patients" or "old age" or "old people" or "middle age" or "middle ages" or menopaus* or "post menopause" or "post menopausal" or postmenopaus*):ti,ab,kw	396963
5.	3 AND 4	1993
**Interventions**		
6.	[mh "orthopedic fixation devices"] or [mh "bone nails"] or [mh "bone plates"] or [mh "bone screws"] or [mh "bone wires"] or [mh "casts, surgical"] or [mh "external fixators"] or [mh "internal fixators"] or [mh splints] or [mh "suture anchors"] or [mh "orthotic devices"] or [mh "athletic tape"] or [mh braces] or [mh "fracture fixation, internal"] or [mh "fracture fixation, intramedullary"] or [mh "fracture fixation"] or [mh arthroplasty]	8318
7.	(nail* or plate or plating or plates or pin or pins or pinning or implant* or screw* or wire* or "k-wire" or kirschner* or splint* or "tension band" or orthotic or orthosis or tape or plaster or brace* or collar or slab or bridging or "non-bridging" or nonbridging or "external fixation" or "internal fixation" or prosthes* or cast or casts or plaster* or arthroplast* or (non next surgical) or (nonsurgical next treatment*) or (conservative next treatment*)): ti,ab,kw	47232
8.	6 OR 7	47852
**Combined sets**		
9.	5 AND 8	1106
**Limits**		
10.	Publication Year from 1990 to 2016	
	**9 AND 10**	**581**<br>CDSR/69<br>DARE/21<br>Central/476<br>CRM/HTA/3<br>EED/12

The search result, usually found at the end of the documentation, forms the list of abstracts.

“ “ = Citation Marks; searches for an exact phrase

CDSR = Cochrane Database of Systematic Review
CENTRAL = Cochrane Central Register of Controlled Trials, “trials”
CRM = Method Studies
DARE = Database Abstracts of Reviews of Effects, “other reviews”
EED = Economic Evaluations

HTA = Health Technology Assessments

Embase via Elsevier 01 December 2016

Title: Treatment options for fractures of the upper extremity in the elderly

Search terms		Items found
**Population: Arm fractures**		
1.	'osteoporosis'/exp/dm_su OR 'fragility fracture'/exp/mj OR 'arm fracture'/exp/mj	25382
2.	((osteoporo* OR fragility OR 'distal radial' OR 'distal radius' OR humerus OR humeral OR humerous OR supracondylar OR radius OR ulna OR wrist* OR "collum chirurgicum" OR hand OR elbow* OR olecranon OR arm OR shoulder* OR 'upper extremities' OR 'upper extremity' OR 'upper limb' OR 'upper limbs' OR forearm* OR 'fore arm' OR barton* OR colle*) NEAR/4 fracture*):ab,ti	43421
3.	1 OR 2	51498
**Population: Age**		
4.	'adult'/de OR 'adulthood'/exp OR 'middle aged'/exp OR 'menopause and climacterium'/exp OR 'aged'/de OR 'aged hospital patient'/exp OR 'frail elderly'/exp OR 'very elderly'/exp OR (adult* OR old OR older):ti OR (aging OR 'middle age' OR 'middle ages' OR middleage* OR menopaus* OR postmenopaus* OR elderly OR elder OR aged OR 'old patients' OR 'old age' OR 'old people' OR 'older people'):ti,ab OR (post NEXT/2 menopaus* OR men OR women):ti,ab	7387386
5.	3 AND 4	30130
**Interventions**		
6.	'orthopedic prostheses, orthoses and implants'/exp/mj OR 'fracture treatment'/exp/mj OR 'plaster cast'/exp OR 'arthroplasty'/de OR 'elbow arthroplasty'/exp OR 'finger arthroplasty'/exp OR 'shoulder arthroplasty'/exp OR 'joint mobilization'/exp/mj	137996
7.	(((nail* OR plate OR plating OR plates OR pin OR pins OR pinning OR implant* OR screw* OR wire* OR "k-wire" OR kirschner* OR splint* OR "tension band" OR orthotic OR orthosis OR tape OR plaster OR brace* OR collar OR slab OR bridging or "non-bridging" or nonbridging) NEAR/8 (fracture* OR fixation* OR locking OR looked)) OR "internal fixation" OR "external fixation" OR prosthes* OR cast OR casts OR plaster* OR arthroplast* OR (surgical NEXT/2 treatment*) OR (nonsurgical NEXT/2 treatment*) OR (conservative NEXT/2 treatment*)):ab,ti	412008
8.	6 OR 7	475682
**Combined sets**		
9.	5 AND 8	18294
**Study types**		
10.	'randomized controlled trial'/exp OR 'observational study'/exp OR 'clinical study'/exp OR 'meta analysis'/exp OR 'meta analysis (topic)'/exp OR 'evaluation study'/exp OR 'pretest posttest control group design'/exp OR random*:ti OR (randomiz* OR randomis*):ab OR (trial* OR cohort* OR observation* OR longitudinal* OR prospective* OR retrospective* OR 'case control' OR prepost* OR 'pre post' OR 'pre test' OR pretest* OR 'post test' OR posttest* OR metaanaly* OR (meta NEXT/1 analy*)):ab,ti OR (program* NEXT/1 evalut*):ab,ti	9540104
**Limits**		
11.	([article]/lim OR [article in press]/lim OR [review]/lim) AND ([danish]/lim OR [english]/lim OR [norwegian]/lim OR [swedish]/lim) AND [1990-2016]/py	
12.	(('animal'/exp OR 'nonhuman'/exp) NOT 'human'/exp)	
	**(9 AND 10 AND 11) NOT 12**	**5102**

/de= Term from the EMTREE controlled vocabulary
/exp= Includes terms found below this term in the EMTREE hierarchy
/mj = Major Topic
:ab = Abstract
:au = Author
:ti = Article Title
:ti:ab = Title or abstract

* = Truncation
“ “ = Citation Marks; searches for an exact phrase “ “ = Citation Marks; searches for an exact phrase

Scopus via Elsevier 02 December 2016

Title: **Treatment options for fractures of the upper extremity in the elderly**

	Search terms	Items found
**Population: Arm fractures**		
1.	TITLE (osteoporo* W/15 fracture*) OR (radius W/15 fracture*) OR (radial W/15 fracture*) OR (distal W/15 fracture*) OR "colles fracture" OR "colles fractures" OR (ulna W/15 fracture*) OR (humeral W/15 fracture*) OR (humerous W/15 fracture*) OR (humerus W/15 fracture*) OR supracondylar OR (shoulder W/15 fracture*) OR (wrist W/15 fracture*) OR (collum W/3 chirurg*) OR (elbow W/15 fracture*) OR olecranon OR barton	25306
**Population: Age**		
2.	TITLE-ABS-KEY (adult* or old or older or aging or elderly or elder or aged or "old patients" or "old age" or "old people" or "middle age" or "middle ages" or menopaus* or "post menopause" or "post menopausal" or postmenopaus*)	9261568
**Interventions**		
3.	TITLE-ABS-KEY (nail* OR plate OR plating OR plates OR pin OR pins OR pinning* OR implant* OR screw* OR wire* OR k-wire OR k-wires OR kirschner* OR splint* OR (tension W/15 band*) OR orthotic OR orthosis OR tape OR plaster OR brace* OR collar OR slab OR bridging OR non-bridging OR nonbridging OR external fixat* OR internal fixat* OR prosthes* OR cast OR casts OR plaster* OR arthroplast* OR "non-surgical treatment" OR "nonsurgical treatment" OR (conservative W/15 treatment*))	2310964
**Combined sets**		
4.	1 AND 2 AND 3	6286
**Study types**		
5.	TITLE-ABS-KEY random* OR cohort* OR longitudinal OR prospective OR retrospective OR observational OR registr* OR "case-control" OR "clinical trial" OR "clinical trials"	4827592
**Limits**		
6.	(LIMIT-TO (DOCTYPE, "ar") OR LIMIT-TO (DOCTYPE,"re")) AND (LIMIT- TO(LANGUAGE, "English", AND (LIMIT-TO(SRCTYPE,"j")	
	**4 AND 5 AND 6**	**1982**

TITLE-ABS-KEY = Title or abstract or keywords
ALL = All fields
PRE/n = "precedes by". The first term in the search must precede the second by a specified number of terms (n).
W/n = "within". The terms in the search must be within a specified number of terms (n) in any order.
***** = Truncation
" " = Citation Marks; searches for an exact phrase

LIMIT-TO (SRCTYPE , "j" = Limit to source type journal
LIMIT-TO (DOCTYPE , "ar" = Limit to document type article
LIMIT-TO (DOCTYPE , "re" = Limit to document type review

Health economic aspects

PubMed via NLM 01 December 2016

Title: Treatment options for fractures of the upper extremity in the elderly: health economic aspects

Search terms		Items found
**Population: Arm fractures**		
1.	"Osteoporotic Fractures"[Mesh] OR "Osteoporosis/surgery"[Mesh] OR "Radius Fractures"[Majr] OR "Colles' Fracture"[Majr] OR "Ulna Fractures"[Majr] OR "Humeral Fractures"[Majr] OR "Shoulder Fractures"[Majr] OR "Wrist Injuries"[Majr] OR humeral[Title] OR distal radial[Title] OR osteoporo*[Title] OR ulna[Title]	49337
2.	(((osteoporo*[Title/Abstract] OR distal radial[Title/Abstract] OR distal radius[Title/Abstract] OR humerus[Title/Abstract] OR humeral[Title/Abstract] OR humerous[Title/Abstract] OR supracondylar[Title/Abstract] OR ulna[Title/Abstract] OR wrist*[Title/Abstract] OR collum chirurg*[Title/Abstract] OR elbow*[Title/Abstract] OR olecranon[Title/Abstract] OR shoulder*[Title/Abstract] OR arm[Title/Abstract] OR upper extremit*[Title/Abstract] OR upper limb*[Title/Abstract] OR forearm*[Title/Abstract] OR fore arm*[Title/Abstract] OR barton*[Title/Abstract] OR colle*[Title/Abstract]) AND fracture*[Title/Abstract]) NOT medline[SB])	7607
3.	1 OR 2	*55029*
**Interventions**		
4.	"surgery" [Subheading] OR "Orthopedic Fixation Devices"[Majr] OR "Bone Nails"[Majr] OR "Bone Plates"[Majr] OR "Bone Screws"[Majr] OR "Bone Wires"[Majr] OR "Casts, Surgical"[Majr] OR "External Fixators"[Majr] OR "Internal Fixators"[Majr] OR "Splints"[Majr] OR "Suture Anchors"[Majr] OR "Orthotic Devices"[Mesh] OR "Athletic Tape"[Mesh] OR "Braces"[Mesh]OR "Fracture Fixation, Internal"[Majr] OR "Fracture Fixation, Intramedullary"[Majr] OR "Fracture Fixation/methods"[Majr] OR "Fracture Fixation/methods"[Mesh] OR "Arthroplasty/methods"[Mesh] OR plate*[Title] OR nail*[Title] OR fixation[Title]	1928926
5.	((nail*[Title/Abstract] OR plate[Title/Abstract] OR plating[Title/Abstract] OR plates[Title/Abstract] OR pin[Title/Abstract] OR pins[Title/Abstract] OR pinning*[Title/Abstract] OR implant*[Title/Abstract] OR screw*[Title/Abstract] OR wire*[Title/Abstract] OR k-wire*[Title/Abstract] OR kirschner*[Title/Abstract] OR splint*[Title/Abstract] OR tension band*[Title/Abstract] OR orthotic[Title/Abstract] OR orthosis[Title/Abstract] OR tape[Title/Abstract] OR plaster[Title/Abstract] OR brace*[Title/Abstract] OR collar[Title/Abstract] OR slab[Title/Abstract] OR bridging[Title/Abstract] OR non-bridging[Title/Abstract] OR nonbridging[Title/Abstract] OR external fixat*[Title/Abstract] OR internal fixat*[Title/Abstract] OR prosthes*[Title/Abstract] OR cast[Title/Abstract] OR casts[Title/Abstract] OR plaster*[Title/Abstract] OR arthroplast*[Title/Abstract] OR non-surgical treatment*[Title/Abstract] OR nonsurgical treatment*[Title/Abstract] OR conservative treatment*[Title/Abstract]) NOT medline[SB])	106115
6.	4 OR 5	2027904
**Combined sets**		
7.	3 AND 6	18105
**Health economic aspects (filter: Edited version of NHS EED)**		
8.	(("Economics"[Mesh:NoExp] OR "Costs and Cost Analysis"[Mesh] OR "Economics, Dental"[Mesh] OR "Economics, Hospital"[Mesh] OR "Economics, Medical"[Mesh] OR "Economics, Nursing"[Mesh] OR "Economics, Pharmaceutical"[Mesh] OR economic*[Title/Abstract] OR cost[Title/Abstract] OR costs[Title/Abstract] OR costly[Title/Abstract] OR costing[Title/Abstract] OR price[Title/Abstract] OR prices[Title/Abstract] OR pricing[Title/Abstract] OR pharmacoeconomic*[Title/Abstract] OR "value for money"[Title/Abstract] OR budget*[Title/Abstract] OR (expenditure*[Title/Abstract] NOT energy[Title/Abstract])) NOT (energy	727962

	cost[Title/Abstract] OR oxygen cost[Title/Abstract] OR metabolic cost[Title/Abstract] OR energy expenditure[Title/Abstract] OR oxygen expenditure[Title/Abstract]))	
**Limits**		
9.	Publication date from 1990/01/01 to 2016/12/31, Swedish, Norwegian, English, Danish.	
	**7 AND 8 AND 9**	**355**

7565
The search result, usually found at the end of the documentation, forms the list of abstracts.

[MeSH] = Term from the Medline controlled vocabulary, including terms found below this term in the MeSH hierarchy
[MeSH:NoExp] = Does not include terms found below this term in the MeSH hierarchy
[MAJR] = MeSH Major Topic
[Title/Abstract] = Title or abstract
[Title] = Title
[AU] = Author
[TW] = Text Word
Systematic[SB] = Filter for retrieving systematic reviews
* = Truncation

Embase via Elsevier 01 December 2016

Title: Treatment options for fractures of the upper extremity in the elderly: health economic aspects

	Search terms	Items found
**Population: Arm fractures**		
10.	'osteoporosis'/exp/dm_su OR 'fragility fracture'/exp/mj OR 'arm fracture'/exp/mj	25382
11.	((osteoporo* OR fragility OR 'distal radial' OR 'distal radius' OR humerus OR humeral OR humerous OR supracondylar OR radius OR ulna OR wrist* OR "collum chirurgicum" OR hand OR elbow* OR olecranon OR arm OR shoulder* OR 'upper extremities' OR 'upper extremi-ty' OR 'upper limb' OR 'upper limbs' OR forearm* OR 'fore arm' OR barton* OR colle*) NEAR/15 fracture*):ab,ti	43421
12.	1 OR 2	51498
**Population: Age**		
13.	'adult'/de OR 'adulthood'/exp OR 'middle aged'/exp OR 'menopause and climacterium'/exp OR 'aged'/de OR 'aged hospital patient'/exp OR 'frail elderly'/exp OR 'very elderly'/exp OR (adult* OR old OR older):ti OR (aging OR 'middle age' OR 'middle ages' OR middleage* OR menopaus* OR postmenopaus* OR elderly OR elder OR aged OR 'old patients' OR 'old age' OR 'old people' OR 'older people'):ti,ab OR (post NEXT/2 menopaus*) OR men OR women:ti,ab	7387386
14.	3 AND 4	30130
**Interventions**		
15.	'orthopedic prostheses, orthoses and implants'/exp/mj OR 'fracture treatment'/exp/mj OR 'plaster cast'/exp OR 'arthroplasty'/de OR 'elbow arthroplasty'/exp OR 'finger arthroplas-ty'/exp OR 'shoulder arthroplasty'/exp OR 'joint mobilization'/exp/mj	137996
16.	(((nail* OR plate OR plating OR plates OR pin OR pins OR pinning OR implant* OR screw* OR wire* OR "k-wire" OR kirschner* OR splint* OR "tension band" OR orthotic OR orthosis OR tape OR plaster OR brace* OR collar OR slab OR bridging or "non-bridging" or nonbridging) NEAR/8 (fracture* OR fixation* OR locking OR looked)) OR "internal fixation" OR "external fixation" OR prosthes* OR cast OR casts OR plaster* OR arthroplast* OR (surgical NEXT/2 treatment*) OR (nonsurgical NEXT/2 treatment*) OR (conservative NEXT/2 treat-ment*)):ab,ti	412008
17.	6 OR 7	475682
**Combined sets**		
18.	5 AND 8	20542
**Health economic aspects (filter: revised version of NHS EED)**		
19.	('health economics'/de OR 'economic evaluation'/exp OR 'health care cost'/exp OR 'phar-macoeconomics'/exp OR econom*:ab,ti OR cost:ab,ti OR costs:ab,ti OR costly:ab,ti OR cost-ing:ab,ti OR price:ab,ti OR prices:ab,ti OR pricing:ab,ti OR pharmacoeconomic*:ab,ti OR (ex-penditure* NOT energy):ti,ab OR (value NEXT/2 money):ab,ti OR Budget*:ab,ti) **NOT** ((metabolic NEXT/2 cost):ab,ti OR ((energy or oxygen) NEXT/2 cost):ab,ti OR ((energy or ox-ygen) NEAR/2 expenditure):ab,ti)	1084700
**Limits**		
20.	([danish]/lim OR [english]/lim OR [norwegian]/lim OR [swedish]/lim) AND [1990-2016]/py	
	**9 AND 10 AND 11**	**316**

/de= Term from the EMTREE controlled vocabulary
/exp= Includes terms found below this term in the EMTREE hierarchy
/mj = Major Topic
:ab = Abstract
:au = Author
:ti = Article Title
:ti:ab = Title or abstract

* = Truncation
“ “ = Citation Marks; searches for an exact phrase “ “ = Citation Marks; searches for an exact phrase

Cochrane Library via Wiley 01 December 2016 (CDSR, DARE & CENTRAL)

Title: Treatment options for fractures of the upper extremity in the elderly: health economic aspects

	Search terms	Items found
**Population: Arm fractures**		
21.	[mh "osteoporotic fractures"] or [mh osteoporosis/PX,SU] or [mh "radius fractures"] or [mh "colles' fracture"] OR [mh "ulna fractures"] OR [mh "humeral fractures"] OR [mh "shoulder fractures"] OR [mh "wrist injuries"]	941
22.	((osteoporo* or "distal radial" or "distal radius" or humerus or humeral or humerous or supracondylar or ulna or wrist* or "collum chirurgicum" or elbow* or olecranon or shoulder* or arm or "upper extremities" or "upper extremity" or "upper limb" or "upper limbs" or forearm* or "fore arm" or barton* or colle*) near/4 fracture*):ti,ab,kw	3051
23.	1 OR 2	3168
**Population: Age**		
24.	[mh^adult] or [mh aged] or [mh "middle aged"] or [mh "aged, 80 and over"] OR [mh "frail elderly"] or [mh menopause] or [mh "age factors"] or (adult* or old or older):ti or (aging or elderly or elder or aged or "old patients" or "old age" or "old people" or "middle age" or "middle ages" or menopaus* or "post menopause" or "post menopausal" or postmenopaus*):ti,ab,kw	396963
25.	3 AND 4	1993
**Interventions**		
26.	[mh "orthopedic fixation devices"] or [mh "bone nails"] or [mh "bone plates"] or [mh "bone screws"] or [mh "bone wires"] or [mh "casts, surgical"] or [mh "external fixators"] or [mh "internal fixators"] or [mh splints] or [mh "suture anchors"] or [mh "orthotic devices"] or [mh "athletic tape"] or [mh braces] or [mh "fracture fixation, internal"] or [mh "fracture fixation, intramedullary"] or [mh "fracture fixation"] or [mh arthroplasty]	8318
27.	(nail* or plate or plating or plates or pin or pins or pinning or implant* or screw* or wire* or "k-wire" or kirschner* or splint* or "tension band" or orthotic or orthosis or tape or plaster or brace* or collar or slab or bridging or "non-bridging" or nonbridging or "external fixation" or "internal fixation" or prosthes* or cast or casts or plaster* or arthroplast* or (non next surgical) or (nonsurgical next treatment*) or (conservative next treatment*)): ti,ab,kw	47232
28.	6 OR 7	47852
**Combined sets**		
29.	5 AND 8	1106
**Limits**		
30.	Publication Year from 1990 to 2016	
	**9 AND 10**	**581**<br>CDSR/69<br>DARE/21<br>Central/476<br>CRM/<br>HTA/3<br>EED/12

The search result, usually found at the end of the documentation, forms the list of abstracts.

" " = Citation Marks; searches for an exact phrase

CDSR = Cochrane Database of Systematic Review
CENTRAL = Cochrane Central Register of Controlled Trials, "trials"
CRM = Method Studies

DARE = Database Abstracts of Reviews of Effects, “other reviews”
EED = Economic Evaluations
HTA = Health Technology Assessments
