# Supplementary material for: Treatment of radius or ulna fractures in the elderly: A systematic review covering effectiveness, safety, economic aspects and current practice
Source: PLoS One. 2019 Mar 28;14(3):e0214362. doi: 10.1371/journal.pone.0214362 (PMC6438530; doi:10.1371/journal.pone.0214362)
Supplement: S2 Appendix — (PDF) [file pone.0214362.s002.pdf]

S2 Appendix.

Publications included in a literature review regarding distal radius fractures, performed by the Swedish Agency for Health Technology Assessment and Assessment of Social Services

Included publications – Effectiveness and complications of treatments

Table of Contents

Included publications – Effectiveness and complications of treatments 1

Randomized controlled trials (31 trials)... 1

Non-randomized controlled trials (10 trials) 5

Included publications – health economic evaluations....................................... 6

Randomized controlled trials (31 trials)

1. Abbaszadegan, H. and U. Jonsson (1990). "External fixation or plaster cast for severely displaced Colles' fractures? Prospective 1-year study of 46 patients." Acta Orthop Scand 61(6): 528-530.

2. Arora, R., M. Lutz, C. Deml, D. Krappinger, L. Haug and M. Gabl (2011). "A prospective randomized trial comparing nonoperative treatment with volar locking plate fixation for displaced and unstable distal radial fractures in patients sixty-five years of age and older." J Bone Joint Surg Am 93(23): 2146-2153.

3. Atroshi, I., E. Brogren, G. U. Larsson, J. Kloow, M. Hofer and A. M. Berggren (2006). "Wrist-bridging versus non-bridging external fixation for displaced distal radius fractures: a randomized assessor-blind clinical trial of 38 patients followed for 1 year." Acta Orthop 77(3): 445-453.

4. Azzopardi, T., S. Ehrendorfer, T. Coulton and M. Abela (2005). "Unstable extra-articular fractures of the distal radius: a prospective, randomised study of immobilisation in a cast versus

supplementary percutaneous pinning." J Bone Joint Surg Br 87(6): 837-840.

5. Bartl, C., D. Stengel, T. Bruckner and F. Gebhard (2014). "The treatment of displaced intra-articular distal radius fractures in elderly patients." Dtsch Arztebl Int 111(46): 779-787.

6. Cassidy, C., J. B. Jupiter, M. Cohen, M. Delli-Santi, C. Fennell, C. Leinberry, J. Husband, A. Ladd, W. R. Seitz and B. Constanz (2003). "Norian SRS cement compared with conventional fixation in distal radial fractures. A randomized study." J Bone Joint Surg Am 85-a(11): 2127-2137.

7. Costa, M. L., J. Achten, N. R. Parsons, A. Rangan, D. Griffin, S. Tubeuf and S. E. Lamb (2014). "Percutaneous fixation with Kirschner wires versus volar locking plate fixation in adults with dorsally displaced fracture of distal radius: randomised controlled trial." Bmj 349: g4807.

8. Foldhazy, Z. and A. Leif (2010) "External fixation versus closed treatment of displaced distal radial fractures in elderly patients: A randomized controlled trial." Current orthopaedic practice 21, 288-295 DOI: 10.1097/BCO.0b013e3181cd6513.

9. Goehre, F., W. Otto, S. Schwan, T. Mendel, P. P. Vergroesen and L. Lindemann-Sperfeld (2014). "Comparison of palmar fixed-angle plate fixation with K-wire fixation of distal radius fractures (AO A2, A3, C1) in elderly patients." J Hand Surg Eur Vol 39(3): 249-257.

10. Gradl, G., G. Gradl, M. Wendt, T. Mittlmeier, G. Kundt and J. B. Jupiter (2013). "Non-bridging external fixation employing multiplanar K-wires versus volar locked plating for dorsally displaced fractures of the distal radius." Arch Orthop Trauma Surg 133(5): 595-602.

11. Gradl, G., N. Mielsch, M. Wendt, S. Falk, T. Mittlmeier, P. Gierer and G. Gradl (2014). "Intramedullary nail versus volar plate fixation of extra-articular distal radius fractures. Two year results of a prospective randomized trial." Injury 45 Suppl 1: S3-8.

12. Hegeman, J. H., J. Oskam, J. Palen, H. J. Duis and P. A. M. Vierhout (2004) "Primary external fixation versus plaster immobilization of

the intra-articular unstable distal radial fracture in the elderly." Aktuelle Traumatologie 34, 64-70 DOI: 10.1055/s-2004-815749.

13. Jakubietz, M. G., J. G. Gruenert and R. G. Jakubietz (2011). "The use of beta-tricalcium phosphate bone graft substitute in dorsally plated, comminuted distal radius fractures." J Orthop Surg Res 6: 24.

14. Jakubietz, M. G., J. G. Gruenert and R. G. Jakubietz (2012). "Palmar and dorsal fixed-angle plates in AO C-type fractures of the distal radius: is there an advantage of palmar plates in the long term?" J Orthop Surg Res 7(1): 8.

15. Kelly, A. J., D. Warwick, T. P. Crichlow and G. C. Bannister (1997) "Is manipulation of moderately displaced Colles' fracture worthwhile? A prospective randomized trial." Injury 28, 283-287.

16. Kim, J. K., Y. D. Koh and S. H. Kook (2011). "Effect of calcium phosphate bone cement augmentation on volar plate fixation of unstable distal radial fractures in the elderly." J Bone Joint Surg Am 93(7): 609-614.

17. Marcheix, P. S., A. Dotzis, P. E. Benko, J. Siegler, J. P. Arnaud and J. L. Charissoux (2010). "Extension fractures of the distal radius in patients older than 50: a prospective randomized study comparing fixation using mixed pins or a palmar fixed-angle plate." J Hand Surg Eur Vol 35(8): 646-651.

18. McFadyen, I., J. Field, P. McCann, J. Ward, S. Nicol and C. Curwen (2011). "Should unstable extra-articular distal radial fractures be treated with fixed-angle volar-locked plates or percutaneous Kirschner wires? A prospective randomised controlled trial." Injury 42(2): 162-166.

19. McQueen, M. M. (1998). "Redisplaced unstable fractures of the distal radius. A randomised, prospective study of bridging versus non-bridging external fixation." J Bone Joint Surg Br 80(4): 665-669.

20. McQueen, M. M., C. Hajducka and C. M. Court-Brown (1996). "Redisplaced unstable fractures of the distal radius: a prospective randomised comparison of four methods of treatment." J Bone Joint Surg Br 78(3): 404-409.

21. Mellstrand Navarro, C., L. Ahrengart, H. Tornqvist and S. Ponzer (2016). "Volar Locking Plate or External Fixation With Optional Addition of K-Wires for Dorsally Displaced Distal Radius Fractures: A Randomized Controlled Study." J Orthop Trauma 30(4): 217-224.

22. Millett, P. J., N. Rushton and P. J. Millet (1995) "Early mobilization in the treatment of Colles' fracture: a 3 year prospective study." Injury 26, 671-675.

23. Sanchez-Sotelo, J., L. Munuera and R. Madero (2000) "Treatment of fractures of the distal radius with a remodellable bone cement." Journal of bone and joint surgery. British volume 82, 856-863.

24. Schmalholz, A. (1990). "External skeletal fixation versus cement fixation in the treatment of redislocated Colles' fracture." Clin Orthop Relat Res(254): 236-241.

25. Schonnemann, J. O., T. B. Hansen and K. Soballe (2011). "Randomised study of non-bridging external fixation compared with intramedullary fixation of unstable distal radial fractures." J Plast Surg Hand Surg 45(4-5): 232-237.

26. Strohm, P. C., C. A. Muller, T. Boll and U. Pfister (2004). "Two procedures for Kirschner wire osteosynthesis of distal radial fractures. A randomized trial." J Bone Joint Surg Am 86-a(12): 2621-2628.

27. Tanaka, H., T. Hatta, K. Sasajima, E. Itoi and T. Aizawa (2016). "Comparative study of treatment for distal radius fractures with two different palmar locking plates." J Hand Surg Eur Vol 41(5): 536-542.

28. Tumia, N., D. Wardlaw, J. Hallett, R. Deutman, S. A. Mattsson and B. Sanden (2003). "Aberdeen Colles' fracture brace as a treatment for Colles' fracture. A multicentre, prospective, randomised, controlled trial." J Bone Joint Surg Br 85(1): 78-82.

29. Vang Hansen, F., H. Staunstrup and S. Mikkelsen (1998). "A comparison of 3 and 5 weeks immobilization for older type 1 and 2 Colles' fractures." J Hand Surg Br 23(3): 400-401.

30. Wong, T. C., Y. Chiu, W. L. Tsang, W. Y. Leung, S. K. Yam and S. H. Yeung (2010). "Casting versus percutaneous pinning for extra-articular fractures of the distal radius in an elderly Chinese population: a prospective randomised controlled trial." J Hand Surg Eur Vol 35(3): 202-208.

31. Zimmermann, R., M. Gabl, M. Lutz, P. Angermann, M. Gschwentner and S. Pechlaner (2003). "Injectable calcium phosphate bone cement Norian SRS for the treatment of intra-articular compression fractures of the distal radius in osteoporotic women." Arch Orthop Trauma Surg 123(1): 22-27.

Non-randomized controlled trials (10 trials)

1. Board, T., A. Kocialkowski and G. Andrew (1999). "Does Kapandji wiring help in older patients? A retrospective comparative review of displaced intra-articular distal radial fractures in patients over 55 years." Injury 30(10): 663-669.

2. Cha, S. M., H. D. Shin, K. C. Kim and E. Park (2012). "Treatment of unstable distal ulna fractures associated with distal radius fractures in patients 65 years and older." J Hand Surg Am 37(12): 2481-2487.

3. Egol, K. A., M. Walsh, S. Romo-Cardoso, S. Dorsky and N. Paksima (2010). "Distal radial fractures in the elderly: operative compared with nonoperative treatment." J Bone Joint Surg Am 92(9): 1851-1857.

4. Lattmann, T., M. Dietrich, C. Meier, M. Kilgus and A. Platz (2008). "Comparison of 2 surgical approaches for volar locking plate osteosynthesis of the distal radius." J Hand Surg Am 33(7): 1135-1143.

5. Navarro, C. M., H. J. Pettersson and A. Enocson (2015). "Complications after distal radius fracture surgery: results from a Swedish nationwide registry study." J Orthop Trauma 29(2): e36-42.

6. Neidenbach, P., L. Audige, M. Wilhelmi-Mock, B. Hanson and P. De Boer (2010). "The efficacy of closed reduction in displaced distal radius fractures." Injury 41(6): 592-598.

7. Oshige, T., A. Sakai, Y. Zenke, S. Moritani and T. Nakamura (2007). "A comparative study of clinical and radiological outcomes of dorsally angulated, unstable distal radius fractures in elderly patients: intrafocal pinning versus volar locking plating." J Hand Surg Am 32(9): 1385-1392.

8. Schmelzer-Schmied, N., P. Wieloch, A. K. Martini and W. Daecke (2009). "Comparison of external fixation, locking and non-locking palmar plating for unstable distal radius fractures in the elderly." Int Orthop 33(3): 773-778.

9. Yamashita, K., Y. Zenke, A. Sakai, T. Oshige, S. Moritani and T. Maehara (2015). "Comparison of Functional Outcome Between Early and Delayed Internal Fixation Using Volar Locking Plate for Distal Radius Fractures." J uoeh 37(2): 111-119.

10. Zenke, Y., A. Sakai, T. Oshige, S. Moritani, Y. Fuse, T. Maehara and T. Nakamura (2011). "Clinical results of volar locking plate for distal radius fractures: conventional versus minimally invasive plate osteosynthesis." J Orthop Trauma 25(7): 425-431.

Included publications – health economic evaluations

1. Tubeuf, S., G. Yu, J. Achten, N. R. Parsons, A. Rangan, S. E. Lamb and M. L. Costa (2015). "Cost effectiveness of treatment with percutaneous Kirschner wires versus volar locking plate for adult patients with a dorsally displaced fracture of the distal radius: analysis from the DRAFFT trial." Bone Joint J 97-b(8): 1082-1089.
