# Supplementary material for: Treatment of radius or ulna fractures in the elderly: A systematic review covering effectiveness, safety, economic aspects and current practice
Source: PLoS One. 2019 Mar 28;14(3):e0214362. doi: 10.1371/journal.pone.0214362 (PMC6438530; doi:10.1371/journal.pone.0214362)
Supplement: S5 Appendix — (PDF) [file pone.0214362.s005.pdf]

S5 Appendix Cost analysis - units and unit costs

Resource utilization for treatment of distal radius fractures in a Swedish setting, estimated for the purpose of a health - economy evaluation performed by the Swedish Agency for Health Technology Assessment and Assessment of Social Services

Resources	Plaster	Plate fixation	Pins	External fixation
Implant/material per treatment	1 roll of plaster, bandage and tape	1 plate with associated screws, bandage, possibly 1 roll of plaster and tape, surgical dressing	3 pins, 1 roll of plaster, bandage and tape, surgical dressing	4 pins and associated reusable pin clamps and couplings, surgical dressing
Preparation time (minutes)[1]	0	60	60	60
Operating time/ treatment time (minutes)	0	70 [1,2]	40 [2,3]	40 [1,4]
Post operation time (minutes)[1]	0	40	40	40
Orthopaedic surgeon (minutes)[2]	40	90	60	60
Assisting orthopaedic	0	35	20	20

surgeon (minutes)[3]				
Anesthesist (minutes) [1]	0	45	45	45
Anaesthetic nurse (minutes) [4]	0	170	140	140
Surgical nurse (minutes)[4]	0	170	140	140
Operation assistant (minutes) [4]	0	170	140	140
Out-patient clinic nurse (minutes) [5]	40	0	0	0
Inpatient care duration due to operation (days)[5]	0	0	0	0

[1] Assumption by expert senior scientists in the research group based on reported data from three large Swedish general hospitals' operation planning systems.
[2] Included during operating time + 10 minutes before and 10 minutes after for preparation and documentation of surgical procedure.
[3] Included only for part of operating time. Represents time needed for training of junior orthopaedic surgeons.
[4] Included during preparation time, operating time and post operation time.
[5] Assumption by expert senior scientists in the research group.

Unit costs for treatment of distal radius fracture in US Dollars, 2016. Estimated for the purpose of a HTA analysis performed by the Swedish Agency for Health Technology Assessment and Assessment of Social Services

Resources for radius fracture treatment	Unit cost per treatment	References
Plaster	6	[6]
Plate fixation with screws	233	[6-8]
Pins (3 pins)	26	[6-8]
External fixation (4 pins and associated reusable pin clamps and couplings)	142	[7,8]
Other resources	Unit cost per minute	References
Theatre operating time (including operating theatre rental, overhead costs and fixed equipment)	3.2	[7]
Orthopaedic surgeon	2.4	[7]
Assisting orthopaedic surgeon	2.4	[7]
Anesthesist	2.4	[7]
Anaesthetic nurse	1.1	[7]
Surgical nurse	1.1	[7]
Operation assistant	0.8	[7]
Out-patient clinic nurse	0.9	[7]

References

1. Navarro CM, Pettersson HJ, Enocson A. Complications after distal radius fracture surgery: results from a Swedish nationwide registry study. Journal of orthopaedic trauma. 2015;29(2):e36-42. Epub 2014/07/23. doi: 10.1097/bot.0000000000000199. PubMed PMID: 25050752.
2. Costa ML, Achten J, Caroline P, Parsons NR, Rangan A, Tubeuf S, et al. UK DRAFFT: A randomised controlled triaof percutaneous fixation with kirschner wires versus volar locking-plate fixation in the treatment of adult patients with a dorsally displaced fracture of the distal radius. Health Technology Assessment. 2015;19(17). doi: 10.3310/hta19170.
3. Pritchett JW. External fixation or closed medullary pinning for unstable Colles fractures? The Journal of bone and joint surgery British volume. 1995;77(2):267-9. Epub 1995/03/01. PubMed PMID: 7706344.
4. Schonnemann JO, Hansen TB, Soballe K. Randomised study of non-bridging external fixation compared with intramedullary fixation of unstable distal radial fractures. Journal of plastic surgery and hand surgery. 2011;45(4-5):232-7. Epub 2011/12/14. doi: 10.3109/2000656x.2011.613243. PubMed PMID: 22150146.
5. Strohm PC, Muller CA, Boll T, Pfister U. Two procedures for Kirschner wire osteosynthesis of distal radial fractures. A randomized trial. The Journal of bone and joint surgery American volume. 2004;86-a(12):2621-8. Epub 2004/12/14. PubMed PMID: 15590845.
6. Operation planning system, Skåne University Hospital, Malmö, Sweden
7. Operation planning system Orbit, Södersjukhuset (Stockholm South General Hospital), Stockholm, Sweden
8. Operation planning system Operätt, Sahlgrenska University Hospital, Gothenburg, Sweden
