# Supplementary material for: Treatment of radius or ulna fractures in the elderly: A systematic review covering effectiveness, safety, economic aspects and current practice
Source: PLoS One. 2019 Mar 28;14(3):e0214362. doi: 10.1371/journal.pone.0214362 (PMC6438530; doi:10.1371/journal.pone.0214362)
Supplement: S6 Appendix — (PDF) [file pone.0214362.s006.pdf]

S6 Appendix Description of studies

Description of studies included in meta-analyses regarding treatment of distal radius and ulna fractures, and an evaluation of the risk of bias of individual studies. All data was collected for the purpose of a literature review performed by the Swedish Agency for Health Technology Assessment and Assessment of Social Services, SBU.

Distal radius fractures

Surgical vs non-surgical treatment

Treatment comparison	Studies (**RCTs** and *cohort studies*), *Fracture type** *Treatment*	n	Age	Outcome measurements	Level of bias	Comments
Plate fixation vs non-operative treatment in plaster	**Arora, 2011** *Distal radius fractures, dorsally displaced without intraarticular displacement. Volar locking plate vs non-operative treatment in plaster*	90	>65 Mean age: 75.9 (65–88)	DASH PRWE Grip strength Complications	Low	
	**Bartl, 2014** *Distal radius fractures, AO type C1–C3. Volar locking plate vs non-operative treatment in plaster*	185	>65	DASH EQ-5D	Moderate	45% cross over to operative group
	Egol, 2010 (cohort) Distal radius fractures, OTA class A, B, C. Volar locking plate or external fixation vs non-operative treatment in plaster	90	>65	DASH Grip strength Complications	Moderate/Low	Retrospective case-control
Percutaneous surgery vs non-operative treatment in plaster	**Abbaszadegan, 1990** *Distal radius fractures, Older type 3 and 4. External fixation vs non-operative treatment in plaster*	47	<75 Mean age: 63 (22–73)	Lidstrom functional grading Grip strength Complications	Moderate	No p-value presented
	**Azzopardi, 2005** *Distal radius fractures, AO type A3 or Frykman type I and II. Percutaneous pinning vs non-operative treatment in plaster*	54	>60 Mean age: 71.5	SF-36 Grip strength Complications	Low	
	Board, 1999 (cohort) Distal radius fractures, AO type C. Percutaneous pinning vs non-operative treatment	46	55-90	Gartland -Werely Complications	Moderate/Low	One surgeon Kapandji technique

	in plaster					
	Egol, 2010 (cohort)<br>*Distal radius fractures, OTA class A, B, C.*<br>*Volar locking plate or external fixation vs non-operative treatment in plaster*	90	>65	DASH<br>Grip strength<br>Complications	Moderate/Low	Retrospective case-control
	**Foldhazy, 2010**<br>*Distal radius fractures dorsally displaced.*<br>*External fixation vs non-operative treatment in plaster*	59	60-85 years	Green O'Brian<br>Grip strength<br>Complications	Low	
	**Hegeman, 2004**<br>*Distal radius fractures, dorsally displaced AO type C2 or C3.*<br>*External fixation vs non-operative treatment in plaster*	32	55-80 years	Gartland –Werely<br>Grip strength<br>Complications	Moderate	
	**McQueen, 1996**<br>*Distal radius fractures dorsally displaced without intraarticular displacement.*<br>*External fixation vs non-operative treatment in plaster*	90	63 years	Grip strength<br>Complications	Low	Study comparing 4 methods of treatment after redisplaced fractures of the distal radius<br>In this comparison bridging fixed external fixation and dynamic external fixation were pooled.
	**Wong, 2010**<br>*Distal radius fractures, extraarticular dorsally displaced.*<br>*Percutaneous pinning vs non-operative treatment in plaster*	62	>65 years<br>Mean age 70.5 (65–76)	Mayo Wrist score<br>WHOQoL<br>Complications	Low	

AO type: Classification of fractures according to Müller ME, Nazarian S, Koch P, 1990, The Comprehensive Classification of Fractures of Long Bones, Berlin, Heidelberg, Springer Verlag. ; **DASH**: Disabilities of the arm, shoulder and hand; **EQ-5D**: EuroQoL 5 Dimensions; **OTA-class**: Orthopedic Trauma Association classification; **PRWE**: Patient Rated Wrist Evaluation;.**RCT**: Randomized Controlled Trial; **SF-36**: 36-item short- **WHOQoL**: World Health Organization Quality of Life. **External fixation is bridging when not otherwise described.**

Surgery vs surgery

Treatment comparison	Studies (**RCTs** and *cohort studies*), *Fracture type* Treatment*	n	Age	Outcome measurements	Level of bias	Comments
Different types of internal fixation with plates	**Jakubietz, 2012** *Distal radius fractures, AO types C1–C3. Volar locking plate vs. dorsal locking plate*	58	>50 Mean age 68 (52–92)	DASH Gartland -Werley Grip strength Complications	Moderate	Hardware removal in all patients at 6 months
	Schmelzer-Schmied, 2009 (cohort) Distal radius fractures, AO types C1–C2. Volar locking plate vs. volar non-locking plate	30	50–70	DASH Complications	Moderate/Low	Part of retrospective matched pair study comparing 3 groups: Volar locking plate (15pats) vs. volar non-locking plate (15 pats) vs external fixation (15 pats)
	Yamashita, 2016 (cohort) Distal radius fractures, extraarticular dorsally displaced. Volar locking plate, primary vs secondary surgery	106	50-80	DASH Grip strength Complications	Moderate/Low	Comparison of 76 pats surgery <1 day vs 30 pats surgery at 7 days
Different types of surgical approaches for internal fixation with volar locking plate	*Lattmann, 2008 (cohort) Distal radius fractures volar or dorsal displacement. Volar locking plate through Henrys incision with or without concomitant carpal tunnel release*	174	Mean age 65 (18-91)	PRWE Grip strength Complications	Moderate/Low	Consecutive patients 2003-2004 (Henrys approach with extended carpal tunnel release) vs 2005-2006 (standard Henry's approach)
	Zenke, 2011 (cohort) Distal radius fractures Dorsally displaced AO types A2, A3, C1, C2. Volar locking plate through Henrys incision vs Minimally Invasive Plate Osteosynthesis technique	66	Mean age 64 (25-94)	DASH Grip strength Complications	Moderate/Low	Retrospective consecutive cohort with prospective data collection.
Different types of percutaneous surgical methods	**Atroshi, 2006** *Distal radius fractures, dorsally displaced without articular step-off. Bridging vs non-bridging external fixation*	38	Females >50, males >60 Mean age 70.5 (55-86)	DASH Grip strength SF-12 Complications	Low	

	**McQueen, 1998**<br>*Distal radius fractures, unstable fractures AO type A3.2, A3.3, C2.1*<br>*Bridging vs non-bridging external fixation*	60	Mean age 61.5	Grip strength<br>Complications	Moderate	
	**Strohm, 2004**<br>*Distal radius fractures AO types A1, A3 and C1*<br>*Percutaneous pinning with Willenegger vs Kapandji technique*	100	Mean age 65 (15-92)	Complications	Low	Functional outcome measured by Martini score not evaluated in meta-analysis
Different types of internal fixation with a plate vs different types of percutaneous surgical methods	**Costa, 2014**<br>*Distal radius fracture, dorsally displaced without comminution of the joint surface.*<br>*Volar locking plate vs percutaneous pinning*	461	>50 years<br>Mean age 59	PRWE<br>DASH<br>EQ-5D<br>Complications	Low	Multicentre. Any dorsally displaced distal radius fracture without comminution of the joint surface
	**Goehre, 2014**<br>*Distal radius fracture, AO types A2, A3 or C1.*<br>*Volar locking plate vs percutaneous pinning*	40	>65 median age 72	DASH<br>Castaing score<br>Grip strength<br>Complications	Moderate	
	**Gradl, 2013**<br>*Distal radius fractures, dorsally displaced, AO types A3, C1, C2 or C3.*<br>*Volar locking plate vs non-bridging external fixation*	102	Mean age: 63 years (18–88)	Gartland- Werley<br>Castaing score<br>Grip strength<br>Complications	Low	
	**Gradl, 2014**<br>*Distal radius fractures, dorsally displaced, AO type A3.*<br>*Volar locking plate vs intramedullary nail*	152	>18 years<br>Mean age 62	Gartland- Werley<br>Castaing score<br>Grip strength<br>Complications	Low	
	**Mellstrand Navarro, 2016**<br>*Distal radius fractures, dorsally displaced, AO-types A2, A3, Cl, C2, and C3.*<br>*Volar locking plate vs external fixation*	140	50 – 74 women, 60–74 men<br>Mean age 63 (50–74)	DASH<br>PRWE<br>EQ-5D<br>Complications	Low	
	Navarro, 2015<br>*Distal radius fraktur*<br>*Plate vs external fixation vs percutaneous pinning*	36618	>18 years<br>Mean age	Complications	Moderate/Low	Registry study of reoperation rates
	Oshige, 2007 Distal radius fractures, dorsally displaced, AO types A2, A3, C1, C2.<br>*Volar locking plate vs percutaneous pinning*	62	>60 (mean age 70 (60–94)	Grip strength	Moderate/Low	Bone Mineral Density presented
	Schmelzer-Schmied, 2009<br>*Distal radius fractures, AO types C1–C3.*<br>*Volar locking plate vs volar non-locking plate vs external fixation*	45	50–70	DASH<br>Complications	Moderate/Low	Part of retrospective matched pair study comparing 3 groups: Volar locking plate

						(15pats) vs. volar non-locking plate (15 pats) vs external fixation (15 pats)

AO type: Classification of fractures according to Müller ME, Nazarian S, Koch P, 1990, The Comprehensive Classification of Fractures of Long Bones, Berlin, Heidelberg, Springer Verlag. ; **DASH**: Disabilities of the arm, shoulder and hand; **EQ-5D**: EuroQoL 5 Dimensions; **PRWE**: Patient Rated Wrist Evaluation; **RCT**: Randomized Controlled Trial; ; **SF-12**: 12-item short-form; **WHOQoL**: World Health Organization Quality of Life. **External fixation is bridging when not otherwise described.**

Non-surgical vs non-surgical treatment

**Treatment comparison**	**Studies** (**RCTs** and *cohort studies*), *Fracture type* Treatment*	**n**	**Age**	**Outcome measurements**	**Level of bias**	**Comments**
Different types of non-surgical treatments	**Millett, 1995** *Distal radius fractures, dorsally displaced. 5 weeks of below the elbow plaster cast vs 3 weeks of below the elbow plaster cast + 2 weeks flexible cast (early wrist mobilization)*	90	Mean age 60.5 (22–88)	Complications	Low	3 years results
	Neidenbach, 2010 (cohort) *Distal radius fractures, AO types 23-A/B/C (except AO 23A-1). Closed reduction and plaster cast vs no reduction and plaster cast*	83	Mean age 62	DASH SF-36 Grip strength	Moderate/Low	62 pats with plaster and closed reduction vs 21 pats with plaster without closed reduction
	**Vang Hansen, 1998** *Distal radius fractures, Older types 1 and 2. 3 weeks of immobilization in plaster cast vs 5 weeks of immobilization in a plaster cast*	100	Mean age 60.5 (18–96)	Grip strength Complications	Moderate	

AO type: Classification of fractures according to Müller ME, Nazarian S, Koch P, 1990, The Comprehensive Classification of Fractures of Long Bones, Berlin, Heidelberg, Springer Verlag. ; **DASH**: Disabilities of the arm, shoulder and hand; **RCT**: Randomized Controlled Trial; **SF-36 (P)**: 36-item short-form

Bone substitute vs. no bone substitute

**Treatment comparison**	**Studies** (**RCTs** and *cohort studies*), *Fracture type* Treatment*	**n**	**Age**	**Outcome measurements**	**Level of bias**	**Comments**
Bone substitute and plating vs no bone substitute and plating	**Jakubietz, 2011** *Distal radius fractures AO type C. Dorsal locking plate with or without bone substitute*	39	>50 (Mean age 67.5)	DASH Gartland Wereley Grip strength Complications	Moderate	All implants removed after 6 months
	**Kim, 2011** *Displaced distal radius fractures. Volar locking plate with or without bone substitute*	50	>65 Mean age 73	DASH Grip strength Complications	Low	

Bone substitute and percutaneous fixation vs no bone substitute and percutaneous fixation	**Cassidy, 2003**<br>*Distal radius fractures, extraarticular, dorsally displaced.*<br>*Closed reduction and percutaneous pinning with bone substitute and plaster vs closed reduction and/or plaster and/or percutaneous pinning and/or external fixation without bone substitute*	323	>45<br>Mean age<br>64	Green O'Brien<br>Grip strength<br>SF-36<br>Complications	Low	
	**McQueen, 1996**<br>*Distal radius fractures dorsally displaced without intraarticular malalignment.*<br>*Open reduction, bone substitute and pinning vs closed reduction and plaster,*	60	61	Grip strength<br>complications	Low	Part of a study comparing 4 groups
	**McQueen, 1996**<br>*Distal radius fractures dorsally displaced without intraarticular malalignment.*<br>*Open reduction, bone substitute and pinning vs vs external fixation*	90	63	Grip strength<br>complications	Low	Part of a study comparing of 4 different treatments after redisplaced fractures of the distal radius groups. Results were pooled for bridging and non-briding external fixation.
	**Sanchez-Sotelo, 2000**<br>*Distal radius fraktur AO types A3 or C2.*<br>*Closed reduction and plaster with bone substitute vs closed reduction and plaster without bone substitute*	110	50-85<br>Mean age<br>66	Green O'Brien<br>Grip strength<br>Complications	Low	
	**Schmalholz, 1990**<br>*Distal radius fractures Frykman types 1 and 2.*<br>*Open reduction, bone substitution and plaster vs closed reduction and external fixation*	48	Mean age<br>66.5 (50-81)	Grip strength<br>Complication	Low	
	**Zimmermann, 2003**<br>*Distal radius fractures AO types C2 or C3.*<br>*Open reduction, screw and pin fixation, bone substitute and plaster vs closed reduction, percutaneous pinning and plaster.*	52	Mean age<br>60 (49-73)	DASH<br>Grip strength<br>Complications	Moderate	

AO type: Classification of fractures according to Müller ME, Nazarian S, Koch P, 1990, The Comprehensive Classification of Fractures of Long Bones, Berlin, Heidelberg, Springer Verlag. ; **DASH**: Disabilities of the arm, shoulder and hand; **RCT**: Randomized Controlled Trial; **SF-36 (P)**: 36-item short-form

Distal ulna fractures

Surgery vs no-surgery

Treatment comparison	Studies (**RCTs** and *cohort studies*), *Fracture type* Treatment*	n	Age	Outcome measurements	Level of bias	Comments
Internal fixation vs non-operative management of distal ulnar fracture	*Cha, 2012*<br>*Ulnar metaphyseal fractures concomitant to plate fixation of distal radius fractures.*<br>*Internal fixation vs non-operative management of the distal ulnar fracture*	61	>64	DASH<br>Grip strength<br>Complications	Moderate/Low	Ulnar metaphyseal fracture within 5 cm of the dome of the ulnar head.

DASH: Disabilities of the arm, shoulder and hand; **RCT**: Randomized Controlled Trial
